# NMR-Based Metabolomic Profiling Highlights Functional Nutritional Gaps Between Human Milk, Infant Formulas, and Animal Milks

**DOI:** 10.3390/metabo15090620

**Published:** 2025-09-18

**Authors:** Gaia Meoni, Leonardo Tenori, Giovanni Niero, Massimo De Marchi, Claudio Luchinat

**Affiliations:** 1 Department of Chemistry “Ugo Schiff”, University of Florence, 50019 Florence, Sesto Fiorentino, Italy; leonardo.tenori@unifi.it; 2 Department of Agronomy, Food, Natural Resources, Animals and Environment, University of Padova, 35020 Padua, Legnaro, Italymassimo.demarchi@unipd.it (M.D.M.); 3 Giotto Biotech S.r.l., 50019 Florence, Sesto Fiorentino, Italy

**Keywords:** NMR, metabolomics, formula milk, milk metabolome, breastfeeding, human milk, goat milk, cow milk, infant nutrition, milk biochemistry

## Abstract

**Background/Objectives:** Human milk represents the gold standard for infant nutrition, providing a complex and bioactive metabolite profile essential for early development. Despite efforts to enrich infant and toddler formulas with functional ingredients, significant biochemical differences persist. This study aimed to characterize and compare the metabolomic composition of human milk, cow’s milk (both conventional and lactose-free), goat’s milk, and a variety of commercial formulas, including both infant (0–12 months) and toddler (1–3 years) formulations, using ^1^H NMR-based metabolomics. **Methods:** A total of 90 milk samples were analyzed, including eight formula milk brands, four human milk samples, four goat milk brands, and seven cow milk products. ^1^H NMR spectra were acquired and processed to identify 54 metabolites. Multivariate and univariate statistical analyses were used to assess compositional similarities and differences among milk types. **Results:** Human milk displayed a unique metabolic signature, enriched in fucosylated oligosaccharides (2′-FL, 3′-FL), N-acetylated carbohydrates, and amino acids such as glutamine and glutamate. Goat milk was characterized by higher levels of creatine, carnitine, and succinate, whereas cow milk showed elevated orotate, butyrate, and mannose. Formulations exhibited a relatively homogeneous composition across brands but differed from human and animal milk, particularly in choline, formate, and added micronutrients. Toddler formulas contained more energy-related compounds (e.g., creatine, maltose) compared to infant formulas. **Conclusions:** While modern formulas provide nutritional adequacy, they remain metabolically distinct from human milk. NMR-based metabolomics offers a powerful tool for guiding future improvements in formula composition toward more biomimetic profiles.

## 1. Introduction

Human milk (HM) is universally recognized as the optimal source of nutrition for infants, offering a dynamic and highly bioactive matrix that supports not only physical growth but also immune system development, gastrointestinal maturation, and neurocognitive function [1,2,3,4]. Its unique composition includes a broad spectrum of essential nutrients, bioactive lipids, free amino acids, nucleotides, and particularly human milk oligosaccharides (HMOs), which play crucial roles in shaping the infant gut microbiota, preventing pathogen adhesion, and modulating immune responses [5,6,7,8]. Among HMOs, 2′-fucosyllactose (2′-FL) and 3′-fucosyllactose (3′-FL) are among the most abundant and have been increasingly studied for their functional relevance in early-life nutrition [9,10,11].

When breastfeeding is not possible or only partially feasible, milk formulas (FMs) are used as alternatives. Over recent years, FMs have evolved to include functional ingredients, with the goal of narrowing the compositional gap with breast milk [12,13]. Despite these innovations, modern FMs still lack the metabolic complexity and inter-individual adaptability of HM, and relatively few studies have comprehensively evaluated how formula products compare to breast milk and other natural milks at the metabolomic level [14,15,16,17]. Animal milks, most commonly cow and, to a lesser extent, goat milk, are typically used as the base for formula production [18]. These milks differ significantly from human milk in their protein composition, oligosaccharide content, and metabolite signatures resulting from ruminant digestion, mammary gland physiology, and feeding practices [19,20]. Goat milk has occasionally been promoted as more similar to human milk in terms of digestibility and allergenicity, yet robust metabolomic comparisons between goat, cow, and human milk remain scarce [21].

This study represents an expansion and refinement of our previous NMR-based metabolomic analysis of FMs and human milk [22].

That initial study focused exclusively on a limited set of five commercial brands and examined only FMs intended for infants and toddlers, primarily contrasting organic versus conventional production systems. While our previous dataset provided valuable insights, such as differences in metabolite levels among different brands, it did not include animal milks and missed the distinctions between the two types of formulations (infant vs. toddler).

The present work expands and refines that approach in several aspects. First, we include natural animal milk (goat milk, lactose-free and conventional cow milk). Second, we introduce new brands and batches of formulas, allowing for a broader and more realistic assessment of the full nutritional landscape, and including products acquired from the market several years after the original study. Third, we extend the comparative framework by analyzing formulas for two developmental stages (0–12 months and 1–3 years), thus enabling the identification of age-specific nutritional adaptations.

High-resolution ^1^H NMR spectroscopy serves as the analytical cornerstone of this work. NMR metabolomics enables simultaneous quantification of diverse small-molecule nutrients and bioactive compounds, including sugars, amino acids, organic acids, and oligosaccharides, in complex matrices such as milk [10,23,24,25]. Its reproducibility and quantitative power make it ideally suited for profiling the metabolic architecture of natural and processed milks, and for detecting both nutrient enrichment and compositional gaps [26]. On this background, the aims of this study are to characterize and compare the metabolomic profiles of human milk, animal milks (cow and goat), and formulas using ^1^H NMR spectroscopy; to assess the degree of metabolic similarity between formulas and breast milk across different brands; and to identify metabolite markers in human milk that may contribute to improving future formulations.

Through this expanded comparative approach, we aim to provide new insights into the biochemical landscape of early-life nutrition and contribute to the optimization of infant feeding strategies.

## 2. Materials and Methods

### 2.1. Study Design and Sample Collection

In this study, we applied ^1^H NMR-based metabolomics to analyze eight commercial formula milk products, covering both infant (0–12 months) and toddler (1–3 years) formulations. Four of these FM brands were newly collected in 2025 and are currently available on the Italian market, while the remaining four had been analyzed in a previous research performed in 2019 [22]. Notably, brand number 4 was included in both datasets, providing two different acquisition time points (2019 and 2025). This expanded dataset enabled a broader comparison across different brands and formulations, and, although based on a limited sample size (number of products analyzed per group), offered insights into potential compositional changes occurring within the same brand over time. Additionally, 4 different brands of goat milk and various types of cow’s milk were collected and analyzed. For cow’s milk, we included 4 different brands of conventional milk and 3 different brands of lactose-free milk. The milk cartons were chosen with a different expiration date, to consider variations due to the different productions. Where possible, more than three cartons have been collected per brand. We also included the NMR spectra of four human milk samples, previously collected [22]. A detailed description of the collection, preparation, and ethical approval procedures is reported in the “Sample Collection” section of the above mentioned publication [22]. Briefly HM samples were obtained from healthy nursing women (one sample per donor) at a mean of 80 ± 68 days (mean/SD) postpartum. One milliliter of each sample was collected by manual expression into a sterile plastic cup and processed for NMR analysis within 120 min of collection. All samples were self-collected and voluntarily donated by breastfeeding women. All products were purchased from retail stores and processed immediately after acquisition. Samples were anonymized by assigning numerical codes to the brands: Brand 1: 5 batches of formula for 0–12 months (each with a different expiration date, as previously described) and 5 batches of formula for 1–3 years; Brand 2: 5 batches of 0–12 months and 5 batches of 1–3 years; Brand 3: 4 batches of 0–12 months and 4 batches of 1–3 years; Brand 4: 5 batches of 0–12 months and 5 batches of 1–3 years collected in 2019; plus 4 batches of 0–12 months and 3 batches of 1–3 years collected in 2025; Brand 5: 5 batches of 0–12 months and 5 batches of 1–3 years; Brand 6: 3 batches of 0–12 months and 3 batches of 1–3 years; Brand 7: 4 batches of 0–12 months and 3 batches of 1–3 years; Brand 8: 4 batches of 0–12 months and 3 batches of 1–3 years.

In addition: Cow milk: 4 different brands of conventional milk (1 batch each) and 3 batches from the same brand of lactose-free milk; Goat milk: 4 different brands (1 batch each); and Human milk: samples from 4 individual donors. In total, 90 milk samples were prepared and analyzed. Table 1 summarizes the samples analyzed.

### 2.2. Sample Preparation and NMR Acquisition

Sample preparation and NMR acquisition protocols were identical to those described in our previous publication [22]. Briefly, each milk sample (700 μL) was mixed with 700 μL of dichloromethane (CH_2_Cl_2_), vortexed, and centrifuged (30 min, 14,000 RCF, 4 °C). The aqueous phase was combined 1:1 with a sodium phosphate buffer (70 mM Na_2_HPO_4_, 20% D_2_O, 6.1 mM NaN_3_, 4.6 mM TSP, pH 7.4), and 600 μL was transferred into 5 mm NMR tubes. ^1^H NMR spectra were acquired at 600.13 MHz using a Bruker Avance spectrometer equipped with a 5 mm PATXI probe and an autosampler (SampleJet). ^1^H NMR spectra were acquired using a standard presaturation pulse sequence to suppress the residual water signal, as the samples were prepared in 20% D_2_O/80% H_2_O. This approach ensured adequate lock performance while minimizing the interference of the H_2_O resonance with metabolite quantification. For each sample 1D ^1^H CPMG (Carr-Purcell-Meiboom-Gill) [27] spectrum was collected to visualize proton signals deriving from small molecules [23], using as acquisition parameters: 73,728 data points, spectral width of 12,019 Hz, 64 scans, relaxation delay (D1) of 4 sec, fixed echo time τ (D20) of 0.3 msec, number of echoes (L4) of 128 and 180° rectangular (hard) pulses.

Spectra were processed using TopSpin 3.8.0 (Bruker, Billerica, MA, USA), applying Fourier transformation, phase/baseline correction, and referencing to the TSP signal at 0.00 ppm. Spectral regions corresponding to residual water (4.57–4.77 ppm) and dichloromethane (5.30–5.65 ppm) were excluded.

### 2.3. Metabolites Identification

Metabolites assignments were carried out using a library of NMR spectra of pure organic compounds (Chenomx NMR Suite 12.0; Edmonton, AB, Canada; https://www.chenomx.com/, accessed on 16 September 2025), supplemented by reference and literature data [10,22,25,26,28,29,30,31,32]. From these signals, a total of 54 metabolites were identified across all milk samples. Peak integration was performed using in-house R (version 4.3.1; https://www.rstudio.com/, accessed on 16 September 2025) script. The table listing the identified molecules is reported as Table S1. Metabolite quantification was performed through integration of representative, well-resolved peaks. In the case of partial signal overlap, only non-overlapping and well-defined resonances were considered for quantification, while signals with significant overlap were excluded to avoid introducing bias.

### 2.4. Statistical Analysis

Statistical analyses were conducted in R software (version 4.3.1).

Principal Component Analysis (PCA) [33] was performed on auto scaled data to explore sample clustering. PC1/PC2 and PC2/PC3 were used for interpretation. The 15 most influential variables were identified based on loading vector magnitude √(PC1^2^ + PC2^2^).

Hierarchical clustering analysis was performed using Euclidean distance and Ward’s method to explore similarities among milk types and formula brands [34]. Prior to clustering, data were normalized using z-score transformation to account for differences in metabolite intensity ranges. Average metabolite values were calculated for each brand to reduce intra-brand variability and facilitate inter-brand comparison.

Metabolite variations between different groups were examined and measured using univariate analyses. The Kruskal–Wallis test followed by Dunn post hoc analysis was chosen to infer significant differences among independent samples from multiple groups (n° groups > 2) [35,36]. The Wilcoxon-Mann–Whitney test was chosen to gather differences between two groups and false discovery rate correction was applied using the Benjamini and Hochberg method (FDR), an adjusted *p*-value < 0.05 was considered statistically significant [37]. To explore and visually compare the distribution of individual metabolites across different experimental groups, we generated boxplots for each quantified compound. The descriptive statistics of the area under the peak (i.e., the integrals of the assigned NMR peaks) were graphically represented using boxplots to illustrate variability across different sample types. Specifically, comparisons were made among infant formula brands (0–12 months), lactose-free cow’s milk, conventional cow’s milk, goat’s milk, and human milk (HM). Boxplots provided a clear overview of the distribution, median, and dispersion of each metabolite within and across groups, highlighting both central tendencies and potential outliers. To visualize the contribution of each formula brand to the relative abundance of different metabolite classes, we generated stacked bar plots grouped by biochemical categories. Metabolites were manually assigned to predefined classes (e.g., amino acids, organic acids, carbohydrates) based on known chemical identity and function. For each metabolite, the mean concentration within each brand was computed and converted into a percentage of the total signal for that metabolite across all brands. These relative contributions were then visualized as stacked bar plots. Dumbbell plots were generated to visualize paired comparisons of metabolite concentrations between infant and toddler formulae across eight commercial brands. For each metabolite, mean and standard deviation values were calculated separately for infant and toddler formulations. To contextualize these values, average concentrations and standard deviations from other milk types (human, cow, goat) were calculated and displayed as background reference bands. The log_2_ fold change (log_2_FC) was computed to evaluate the magnitude and direction of variation in metabolite levels between two groups: HM and the other milk types. For each metabolite, the median integral value (area under the NMR peak) was calculated separately within each group.

## 3. Results

### 3.1. Multivariate Analyses

**Figure 1:**
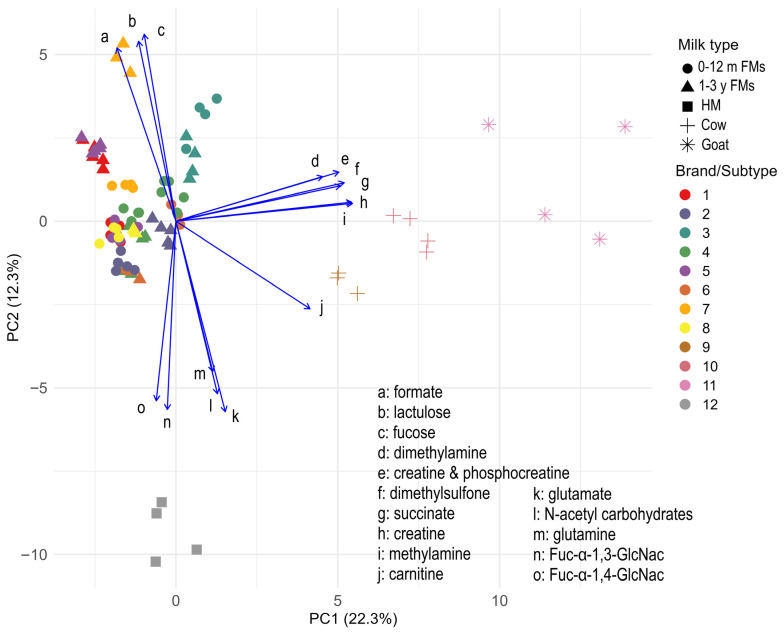
Biplot of the PCA based on the metabolic profiles of different milk types (formulas 0–12 months and 1–3 years are numbered from 1 to 8, human milk is numbered as group 12, cow’s milk 10, lactose-free cow’s milk 9, and goat’s milk 11). The symbols distinguish the types of milk analyzed. The circle symbol corresponds to the milk formulas for newborns 0–12 months, the triangle corresponds to the formulas for 1–3 year old children, the square represents human breast milk samples, the cross represents cow’s milk, and the asterisk represents goat’s milk. The different colors reflect the different milk brands/subtypes (formulas numbered from 1 to 8, lactose-free cow’s milk number 9, conventional cow’s milk number 10, goat’s milk number 11 and human breast milk number 12). The blue arrows represent the loadings of metabolites measured by NMR, indicating the direction and contribution of each compound to the variance explained by PC1 and PC2. Letters closed to the arrows refers to the 15 most influential metabolites (variables). The % of variance explained by each component is reported on the axes.

A PCA was carried out to explore patterns and variability in the NMR profiles of the different milk types. Figure 1 shows a biplot from a PCA performed on the metabolic profiles of milk samples. The first two principal components (PC1 and PC2) explained 22.3% and 12.3% of the total variance, respectively. Animal milks are distributed along the positive axis of PC1 (PC1+), primarily driven by elevated levels of creatine, creatinine and phosphocreatine, dimethylamine, methylamine, dimethyl sulfone, and succinate. These metabolites indicate a profile enriched in compounds associated with protein, energy, and lipid metabolism, features consistent with the nutritional composition of ruminant milk. Goat milk samples (pink asterisks, PC1+) are distinctly separated from all other groups, while cow milk samples (yellow and orange crosses) are more spread along the PC1+/PC2- quadrant, characterized by higher levels of carnitine. Human milk samples (gray squares, PC1-/PC2-) occupied a unique metabolic space, characterized by higher levels of glutamine, glutamate, N-acetylcarbohydrates, and human-specific fucosylated oligosaccharides (Fucosyl-α1,4-N-acetylglucosamine, Fucosyl-α1,3-N-acetylglucosamine). Formulas for children aged 1–3 years (yellow, red, and purple triangles) occupy the upper left quadrant, distinguished by higher levels of formate, fucose and lactulose. Figure S1, reporting PC2 vs. PC3 (explained variance: 11.4%), further highlighted the separation between milk types and formulas. Goat’s milk and formula 1–3y of brand 7 (dark yellow triangles) were characterized by high lactose, histidine, valine and inosine levels, suggesting a metabolic profile tailored for older children and reflecting species-specific milk composition. Cow’s milk (PC2+/PC3+) was rich in orotate and mannose, metabolites indicative of ruminant metabolism and potential intestinal health benefits. Formulas with high formate, fucose, and lactulose were positioned distinctly, suggesting the impact of industrial modifications on their metabolic profiles. Human milk samples aligned horizontally with 0–12 month formulas along PC3, reflecting the compositional adaptation of infant formulas to mimic human milk during early lactation. This figure also provides insight into compositional differences between infant and toddler formula types across different brands. As shown in Figure S1, some brands, such as brand 2 and brand 3, display minimal metabolic variation between their 0–12 months and 1–3 years formulations. In contrast, other brands, particularly brand 1, brand 5, and especially brand 7, exhibit marked differences between the two formulations. Representative ^1^H NMR spectra of the main milk groups are provided in Supplementary Material as Figure S2.

**Figure 2:**
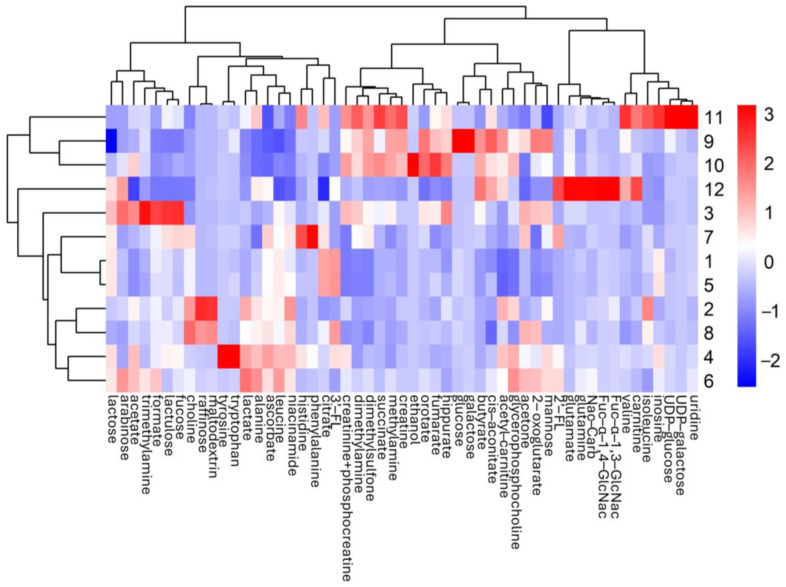
Heatmap of the z-score values of metabolites identified by NMR in the different milk samples analyzed (in this case, the formulas 0–12 months, groups 1–8, human milk, group 12, lactose-free cow’s milk, group 9, cow’s milk 10, and goat’s milk, group 11). The rows represent the milk categories (1–12), while the columns correspond to the metabolites. The color scale varies from blue (negative z-score values) to red (positive z-scores), representing values below or above the global mean of the respective metabolite. The distance between groups (rows) and metabolites (columns) was calculated using Euclidean distance, and hierarchical clustering was performed with the Ward. D2 agglomeration method to generate dendrograms representing the similarity of the average profiles of the brands.

The hierarchical clustering further highlighted the differences and similarities between milk types, reinforcing the PCA findings. Separate analyses were conducted to first compare 0–12 month formulas with human and animal milks (Figure 2), and then follow-on formulas (1–3 years) with animal milks (Figure S3). This approach was chosen because only the 0–12 month formulas are directly comparable to human milk, which was collected from healthy mothers (one sample each) at a mean of 80 ± 68 days postpartum. Moreover, 0–12 month formulas are intended as mostly the sole source of infant nutrition, unlike 1–3 years formulas, which are used alongside solid foods during weaning.

In Figure 2, formulas (0–12 months, groups 1–8) formed a compact cluster, reflecting high compositional similarity likely due to industrial standardization. These formulas were generally enriched in sugars and amino acids, with formula 3 displaying unique metabolic fingerprints, marked by elevated sugars like fucose, lactulose, lactose, mannose, arabinose, and nitrogenous derivatives (trimethylamine, dimethylamine, creatinine, phosphocreatine, hippurate). Human milks (group 12) belong to the same cluster of FMs, occupying and intermediate position between formula milk and animal milk (sharing with animals mostly butyrate, cis-aconitate and acetyl-carnitine). Lactose-free cow’s milk (group 9) and conventional cow’s milk (group 10) are part of the animal cluster, with lactose-free milk showing altered sugar profiles consistent with enzymatic hydrolysis. Cow’s milk was characterized by lower levels of many energy metabolites and a distinct profile of amino acids, and bioactive compounds, reflecting its ruminant origin. Goat’s milk (group 11) occupies a distinct position mostly characterized by higher levels of uridine, UDP-galactose, UDP-glucose, inosine, isoleucine, carnitine and valine.

Figure S3, focused on formulas for 1–3 years and animal milks, confirmed the homogeneity among formulas. This figure highlight group 7 for its unique profile, resulting particularly enriched in several metabolites, including 2′-FL, leucine, tryptophan, isoleucine, phenylalanine, valine and histidine. Its profile deviates significantly from both the other formulas and animal milk, suggesting a different production strategy or targeted composition. The other formula groups generally show intermediate or low values (blue/blue tones) for many metabolites, but with some internal variations due to differences between brands (e.g., 3′-FL, Fucosyl-α1,4-N-acetylglucosamine, Fucosyl-α1,3-N-acetylglu, ascorbate, fucose, lactulose and other sugars). Goat milk, in this case, displays intermediate patterns between cow’s milk and formulas.

### 3.2. Univariate Analyses of Different Milk Types

For a detailed view of metabolite levels in the various groups under investigation, univariate analyses were also performed. Table S2 reports the means and standard deviations for all the milk formulas (0–12 months), lactose-free cow’s milk, conventional cow’s milk, goat’s milk, and human milk. To enhance descriptive clarity, metabolites were grouped according to the biochemical class: amino acids, organic acids, amines and derivatives, vitamins, carbohydrates and sugars, energetic compounds, short chain fatty acids -SCFAs, nucleotides and derivatives, other compounds.

**Figure 3:**
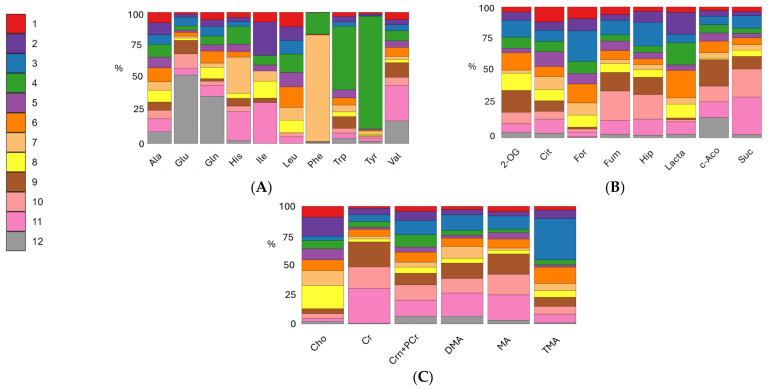
Stacked bar plots representing the relative contribution of each milk type to the total mean (as the % of the class mean) concentration of selected metabolites. For each metabolite, the percentage contribution of each milk class [formulas 0–12 months (numbered from 1 to 8), lactose-free cow’s milk colored in brown (n° 9), conventional cow’s milk colored in salmon pink (n° 10), goat’s milk colored in pink (n° 11), and human milk colored in gray (n° 12)] was calculated as the percentage of mean concentration within the class divided by the sum of means across all classes. Panels show metabolite classes: (**A**) amino acids (alanine: Ala; glutamine: Gln; glutamate: Glu; histidine: His; isoleucine: Ile; leucine: Leu; phenylalanine: Phe; tryptophan: Trp; tyrosine: Tyr; valine: Val), (**B**) organic acids (2-oxoglutarate: 2-OG; cis-aconitate: c-Aco; citrate: Cit; formate: For; fumarate: Fum; hippurate: Hip; lactate: Lacta; succinate: Suc), and (**C**) amines and derivatives (choline: Cho; creatine: Cr; creatinine + phosphocreatine: Crn + PCr; dimethylamine: DMA; methylamine: MA; trimethylamine: TMA).

The stacked bar plots of amino acids (alanine, glutamate, glutamine, histidine, isoleucine, leucine, phenylalanine, tryptophan, tyrosine, valine) are reported in Figure 3A and Figure S4, Table S3 present the boxplots and FDR *p*-values for comparisons of amino acid levels in the 0–12 months formula groups, lactose-free cow’s milk, conventional cow’s milk, goat’s milk, and human milk. Human milk was characterized by significantly higher glutamate and glutamine concentrations. Glutamate, the main excitatory neurotransmitter in the brain, plays a critical role in neuronal development, while glutamine is essential for gut health and immune function. Leucine was essentially absent in human milk but present in conventional cow’s milk, goat’s milk and formulas, whereas valine levels were higher in human and goat milk. Formula 7 showed histidine levels comparable to goat’s milk, and selective enrichment of phenylalanine and tyrosine in formulas like 7 and 4 reflected targeted supplementations. In natural milks, some amino acids are probably part of complex proteins and are thus not detectable in the acquired metabolic profiles.

For organic acids detected (2-oxoglutarate, cis-aconitate, citrate, formate, fumarate, hippurate, lactate, and succinate), as reported in the stacked bar plots of Figure 3B, formate was almost undetectable in human and animal milks but present in formulas, supporting its role as a marker of industrial processing. Hippurate and succinate were more abundant in animal milks, consistent with ruminant metabolism and dietary influences. In general, human milk seems to be characterized by lower amounts of such organic acids with respect to formulas and animals’ milk. Table S4 and Figure S5 reports the FDR *p*-values and boxplots for group comparisons in the organic acids class.

Figure 3C describes the % amount of amines and derivatives (choline, creatine, creatinine and phosphocreatine, dimethylamine, methylamine and trimethylamine). Among amines and derivatives, choline was highest in formulas, aligning with nutritional guidelines for infant formula fortification. Creatinine, phosphocreatine, and methylamine were more abundant in animal milks, supporting their role in muscle and energy metabolism. Creatine levels were abundant in cow and goat milk. Table S5 and Figure S6 report the FDR *p*-values and boxplots for group comparisons in the amines and derivative class.

**Figure 4:**
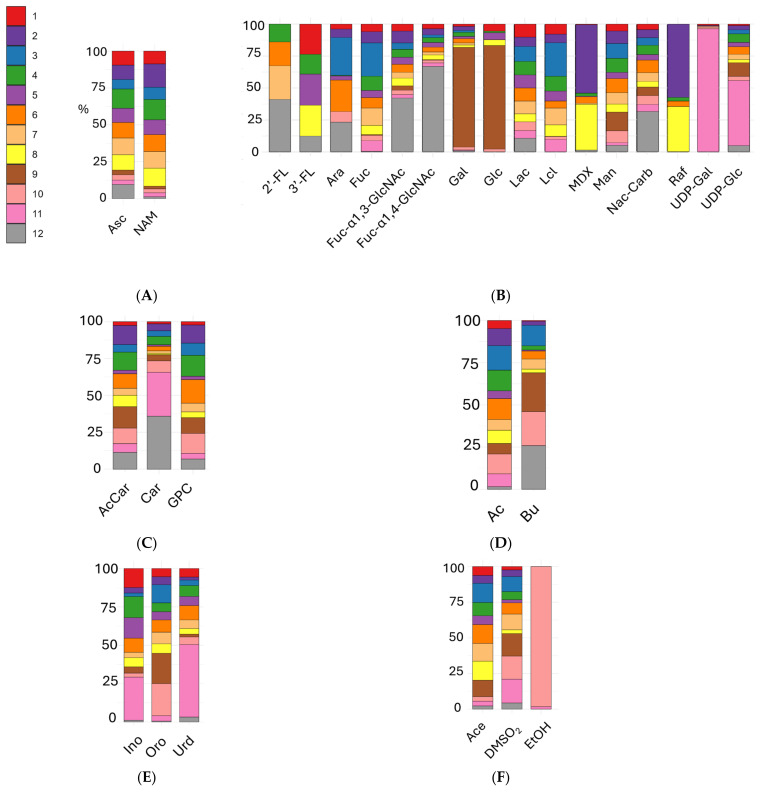
Stacked bar plots representing the relative contribution of each milk type to the total mean concentration (as the % of the class mean) of selected metabolites. Panels show metabolite classes: (**A**) vitamins (ascorbate: Asc; niacinamide: NAM), (**B**) carbohydrates and sugars (2′-fucosyllactose: 2′-FL; 3′-fucosyllactose: 3′-FL; arabinose: Ara; fucose: Fuc; Fucosyl-α-1,3-N-acetylglucosamine: Fuc-α1,3-GlcNAc; Fucosyl-α-1,4-N-acetylglucosamine: Fuc-α1,4-GlcNAc; galactose: Gal; glucose: Glc; lactose: Lac; lactulose: Lcl; maltodextrin: MDX; mannose: Man; N-acetyl carbohydrates: NAc-Carb; raffinose: Raf; sucrose: Suc; UDP-galactose: UDP-Gal; UDP-glucose: UDP-Glc), (**C**) energetic compounds (acetyl-carnitine: AcCar; carnitine: Car; glycerophosphocholine: GPC), (**D**) short chain fatty acids (acetate: Ac; butyrate: Bu), (**E**) nucleotides and derivatives (inosine: Ino; uridine: Urd; orotate: Oro), and (**F**) other compounds (acetone: Ace; dimethylsulfone: DMSO_2_; ethanol: EtOH).

The water-soluble vitamins identified, ascorbate (vitamin C) and niacinamide (vitamin B3) displayed distinct distribution patterns across milk types (Figure 4A). Ascorbate levels were higher in human milk and infant formulas compared to both cow and goat milk. This difference likely reflects the reliance of humans on dietary vitamin C, which is directly mirrored in the composition of breast milk. In contrast, formulas are typically fortified to meet the nutritional requirements of infants. Niacinamide levels were also elevated in formulas compared to human and animal milks, suggesting systematic fortification practices in the production of infant formulas. This is supported by statistical comparisons (Table S6 and Figure S7), which provide FDR-corrected *p*-values and boxplot visualizations of these differences.

Analysis of carbohydrates and sugars (Figure 4B) revealed that human milk exhibits a unique signature. The oligosaccharides 2′-FL, and fucosylated N-acetyl-glucosamines (α 1–3 and α 1–4 linked) were found almost exclusively in human milk, with lower levels in formulas and animal milks. In particular, 2′-FL is present in only three out of the eight infant formulas (0–12 months) analyzed. As shown in the 2′-FL boxplot in Figure S8, Brand 4 exhibits variability in 2′-FL content: this oligosaccharide is detected only in the new batches collected in 2025, while it is absent in the older formulation. Notably, brands 1, 4, 5, and 8 contain 3′-FL at levels comparable to human milk, whereas this oligosaccharide is absent in all other formulas and animal milks (Figure S8). Fucose levels were very low in human milk but relatively high in many formulas and in both cow and goat milk, suggesting differences in sugar sources or metabolic processing. Lactose-free cow’s milk showed the highest abundance of free glucose and galactose, which were also present in formulas, though at lower concentrations. Lactose levels were similar between human milk and formulas, but, as expected, absent in lactose-free milk. Several infant formulas contained maltodextrins and raffinose, not detected in human milk, indicating the use of added polysaccharides and prebiotics during manufacturing. UDP-galactose and UDP-glucose were dominant in goat’s milk, possibly reflecting species-specific differences in nucleotide-sugar metabolism or mammary gland secretion. While some carbohydrate components are shared among matrices, the presence of specific oligosaccharides and metabolic sugar intermediates clearly distinguishes human milk from animal milks and formulas. Table S7 provides statistical details for these comparisons.

Among the energetic compounds (Figure 4C), distinct differences were observed across milk groups (see also Figure S9A and Table S8). Acetyl-carnitine and glycerophosphocholine showed variable levels among formulas and animal milk, whereas goat milk and human milk exhibited higher levels of carnitine compared to both FMs and cow’s milk.

Regarding SCFAs (Figure 4D and Figure S9B, Table S8), human milk displayed significantly lower levels of acetate than all animal milks and formulas. Notably, butyrate levels were almost undetectable in goat milk, low in formulas, and higher in cow’s milk, while they varied widely among individual human mothers.

In the group of nucleotides and derivatives (Figure 4E and Figure S9C, Table S8), inosine and uridine were more abundant in goat milk compared to formulas, cow’s milk, and human milk. Conversely, orotate was significantly elevated in cow’s milk, while being mostly absent in human milk and present at lower concentrations in formulas and goat milk.

For the group of uncategorized compounds (other compounds, Figure 4F and Figure S9D and Table S8), human milk, goat milk, and normal cow’s milk showed consistently lower levels of acetone compared to formulas. Dimethylsulfone was detected at low levels in certain formulas and in human milk but was present at higher concentrations in animal milks. Ethanol was absent in formulas and human milk, while it was variable and generally elevated in regular cow’s milk and very low in goat milk.

### 3.3. Univariate Analyses of Milk Formulas and Human Milk

**Figure 5:**
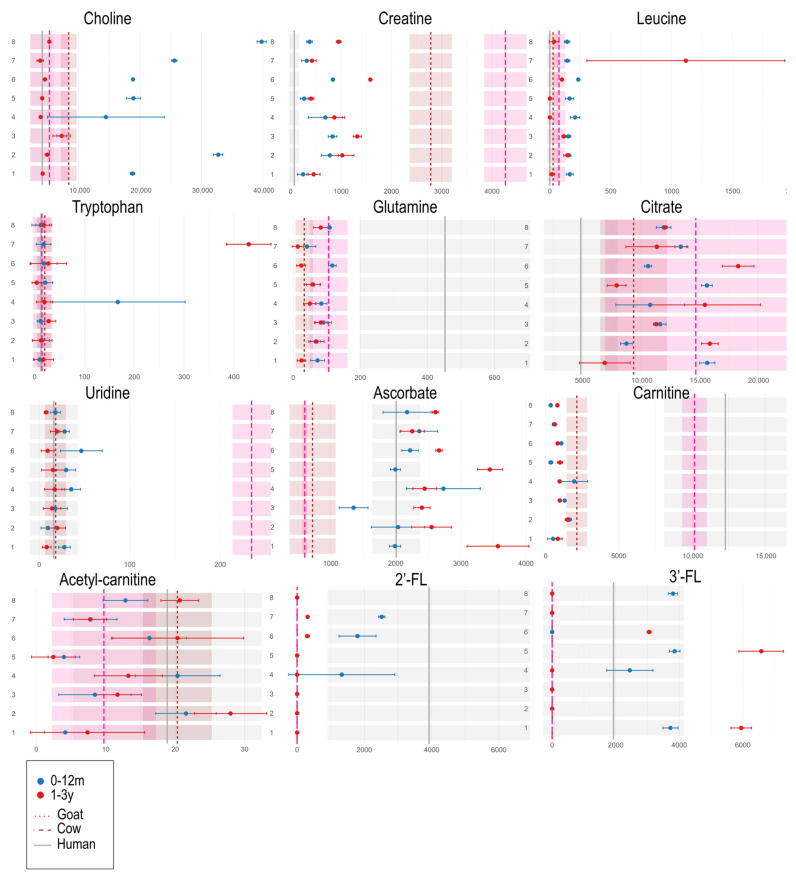
Dumbbell plot. The plots were constructed using the means and standard deviations calculated for each milk type group. On the y-axis, we have the 8 brands of artificial formulas. The blue dots represent the 0–12m type for each FM (formula milk), while the red dots represent the 1–3y type. On the x-axis, we find the calculated peak areas for each signal assigned to a specific metabolite, i.e., the relative intensities of each metabolite. The vertical lines on the plot indicate the group means for goat milk (dashed pink line), cow milk (both lactose-free and conventional, dashed brown line), and human milk (solid gray line). Standard deviations (SD) were also calculated for these and are represented by shaded bands in the same color as the corresponding line. This was done to better describe the trends of the different groups.

The comparative analyses between the two formulation types (0–12 months vs. 1–3 years) reveal that the metabolic trends across different brands are neither symmetrical nor consistent. For clarity and brevity, we focus here on a selection of key metabolites involved in early childhood development (Figure 5; however, full results are available in the Supplementary Material (Table S9, Figures S10 and S11). As shown in the dumbbell plot for choline in Figure 5, 0–12 month formulations are particularly enriched in this compound compared to both animal milks (goat and cow) and human milk (4063.5 ± 1938.1, mean ± SD, see Table S9). Creatine, which functions as an energy reservoir for high-turnover tissues (e.g., brain, muscles), shows higher levels in the 1–3 year formulations compared to the 0–12 month ones. While human milk is relatively poor in creatine, both cow and goat milk are particularly rich in this metabolite. Leucine, an essential amino acid crucial for muscle and neural protein synthesis, is generally more abundant in 0–12 month formulations, except for brand 7. Despite some batch variability, this brand shows a reversed trend (1–3y > 0–12m), with notably higher leucine levels in the 1–3 year formulation. The dumbbell plot for leucine in Figure 5 shows a large standard deviation for the 1–3y group of brand 7, which, corroborated by the NMR spectra in Figure S12, appears to stem from the limited sample size rather than actual compositional diversity. Nonetheless, the trend is evident: both the dumbbell plots and the raw NMR spectra clearly show that the 1–3y formulation of brand 7 contains significantly higher levels of isoleucine, leucine, and valine (see Figure S10). Tryptophan levels are generally very low across all milk types, often close to undetectable. Only a few formulations, such as brand 4 (0–12m) and brand 7 (1–3y), show markedly higher concentrations. As shown in Figure S13, only the 0–12 m formulations of brand 4 collected and analyzed in 2019 contained high tryptophan levels. The same brand’s 2025 samples show nearly no trace of this amino acid, indicating that the formulation has likely been modified over the years, significantly reducing its tryptophan content. Glutamine, like glutamate, is predominantly found in human milk, albeit with considerable variability (Figure 5 and Figure S10). In general, glutamine levels are higher in 0–12 month formulations, although the differences are often marginal both between formulations and across brands. Artificial formulations tend to fall within the intermediate range of glutamine levels found in goat and cow milk (Figure 5). Citrate, a key metabolite in energy metabolism, shows high variability across human and animal milk. Among formula products, there is no clear trend distinguishing the two formulation types. Uridine, which plays a role in synaptic plasticity and brain development, is especially abundant in goat milk. Among formula types, uridine tends to be more concentrated in infant formulations (0–12m) than in toddler ones (1–3y), though overall levels across all formulations remain within the range found in human and cow milk. As for ascorbate (vitamin C), the levels in formula milks closely resemble those of human milk, suggesting that vitamin C is supplemented to mimic the natural profile. In general, 1–3y formulations contain slightly higher ascorbate levels than 0–12m formulations. Carnitine, a metabolite important for brain development, is present in human and animal milk as free L-carnitine, and in esterified form as acetyl-carnitine. As shown in Figure 5, free carnitine is more abundant in human and goat milk, and lower in cow milk and formula products, with no distinct pattern between infant and toddler formulations. On the other hand, acetyl-carnitine levels in formulas are generally closer to those found in human and cow milk. The levels of 2′-FL and 3′-FL vary during the stages of lactation in humans: 2′-FL typically decreases over time, while 3′-FL increases. These oligosaccharides are absent in animal milk but are added to some infant formulas. As shown in the dumbbell plots (Figure 5), 2′-FL levels are, as expected, higher in formulas designed for 0–12 months compared to those for 1–3 years. Only 3 out of the 8 analyzed infant formulas contain added 2′-FL. Notably, Brand 4 includes 2′-FL only in the 2025 batch, while it was absent in the 2019 formulation (this may suggest a recent reformulation aimed at more closely mimicking the oligosaccharide profile of human milk). Regarding 3′-FL, as expected, given that our human milk samples span only the first five months postpartum, formulas for 1–3 years that include this oligosaccharide show higher levels of 3′-FL. In contrast, 3′-FL levels in the 0–12 month formulas that contain it fall within the range observed in human milk.

**Figure 6:**
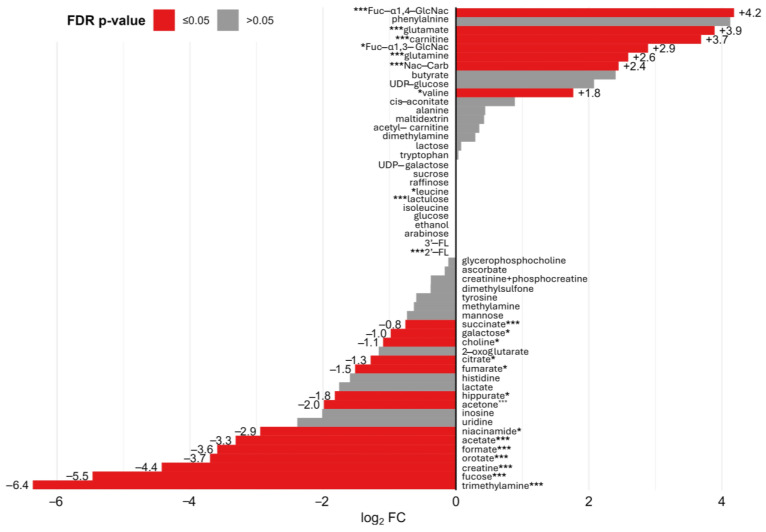
Log_2_ fold change (log_2_(FC)) of median metabolite concentrations in all milk samples compared to the median values in human milk. Positive Log_2_(FC) bars indicate higher metabolite levels in human milk, while negative values indicate lower levels in human milk relative to other milk types (formulas, cow’s milk, and goat’s milk). Metabolites with statistically significant differences (FDR-adjusted *p*-value ≤ 0.05) are shown in red; non-significant metabolites are shown in gray. FDR-adjusted *p*-value: * ≤ 0.05; *** ≤ 0.005.

Finally, the metabolic profile of human milk was evaluated in comparison with all other types of milk included in the study (i.e., all infant formulas, cow milk, and goat milk) to provide a comparative description of the uniqueness of this nutritional source. To this end, a log_2_ fold change (Log_2_(FC)) analysis was performed by dividing the median metabolite concentrations of each milk type by the corresponding median values of human milk samples (Figure 6). Human milk was characterized significantly higher levels of Fuc-α1,4-GlcNAc, glutamate, carnitines, Fuc-α1,3-GlcNAc, glutamine, N-acetyl carbohydrates, and valine. Compared to other matrices, human milk exhibited significantly greater levels of Fuc-α1,4-GlcNAc, glutamate, carnitines, Fuc-α1,3-GlcNAc, glutamine, N-acetyl carbohydrates, and valine, while showing markedly lower concentrations of trimethylamine, fucose, creatine, orotate, formate, acetate, niacinamide, acetone, hippurate, fumarate, citrate, choline, galactose, and succinate.

## 4. Discussion

Our multivariate analyses clearly revealed distinct metabolic signatures across milk types. Human milk formed a well-defined cluster, driven by its richness in fucosylated oligosaccharides such as 2′-FL, 3′-FL, Fuc-α-1,3- and 1,4-N-acetylglucosamine. 2′-FL and 3′-FL among the most abundant HMOs [5,6,11,32]. These findings align with the known dynamics of HMOs in human milk, where 2′-FL tends to decrease and 3′-FL increase over the course of lactation [38]. The variation of 2′-FL and 3′-FL levels across formula types appears to reflect these physiological patterns. In particular, 2′-FL was found in higher amounts in formulas intended for 0–12 months, while 3′-FL was more abundant in toddler (1–3 years) formulas, consistent with the developmental trajectory observed in human milk. Furthermore, our observation that brand 4 only included 2′-FL in its 2025 formulation, and not in the 2019 version, suggests a recent reformulation aimed at more closely mimicking the natural composition of human milk. This supports the broader trend reported in the literature, where infant formulas are increasingly supplemented with HMOs to narrow the compositional gap with human milk.

Along with Fuc-11-1, 3/4-GlcNAc, they are involved in probiotic and antimicrobial functions, modulation of the immune response, and cognitive development in newborns [5,39,40,41,42,43]. The presence of N-acetylated carbohydrates further enhances the gut health by promoting the colonization by beneficial microbes [44,45]. Our results also corroborate the fact that human milk is rich in glutamine and glutamate, amino acids that support immune function and gastrointestinal health [46,47,48]. Together, these components nurture the newborn’s developing gut and contribute to neurocognitive maturation by supporting cellular processes in the brain [49,50].

Animal milks, including both cow’s and goat’s milk, formed a separate cluster in the PCA space, reflecting higher concentrations of creatine, phosphocreatine, carnitine, succinate, and methylamine, metabolites associated with muscular energy metabolism and protein turnover [21,51,52,53,54]. Both conventional and lactose-free cow milk were rich in orotate, mannose, and butyrate, metabolites typically associated with microbial fermentation in the ruminant digestive system. These markers indicate active rumen microbial metabolism and nucleotide biosynthesis [25]. The presence of orotate, a key intermediate in the pyrimidine pathway, and mannose, indicate the microbial component characteristic of ruminants in animal milk production [55]. These metabolic distinctions reflect known physiological and nutritional differences between goats and cows. Goat milk’s elevated creatine and carnitine likely support energy-rich milk composition suitable for neonates, while cow milk’s fermentation-derived metabolites underscore ruminant digestive processes. Seasonal, dietary, and genetic factors can further modulate these metabolic profiles [56].

Notably, animal’s milk samples showed low levels or absence of fucosylated oligosaccharides and had a lower content of free amino acids compared to human milk.

This compositional gap has important functional implications: fucosylated oligosaccharides play key roles in shaping the infant gut microbiota, preventing pathogen adhesion, and modulating immune and cognitive development, while free amino acids act as bioactive molecules supporting metabolism, gut integrity, and immune regulation.

Together, these differences highlight the unique adaptation of human milk to infant needs and provide a rationale for the supplementation of formulas with selected oligosaccharides (e.g., 2′-FL) and amino acids to narrow the functional gap with human milk. The overall lower abundance of free amino acids detected in human milk compared to infant formulas can be attributed to its lower total protein content and to differences in protein composition and digestion. Human milk contains approximately 8–10 g/L of protein in mature milk, which is significantly lower than the protein levels found in most formulas [3,4,38]. However, this does not imply a nutritional deficiency, as the proteins in human milk (particularly α-lactalbumin, lactoferrin, and secretory IgA) are highly digestible and biologically active, supporting immune system, mineral absorption, and gut development [3]. Moreover, a substantial portion of nitrogen in human milk exists as non-protein nitrogen compounds (such as urea, peptides, and nucleotides), which are less prevalent in infant formulas. This difference may account for the greater total protein content required in artificial formulations to satisfy the amino acid needs of infants [56].

Despite the lower overall amino acid levels, human milk showed a higher relative abundance of glutamine and glutamate. These amino acids are critical for neonatal development, serving as major energy substrates for enterocytes and playing roles in gut integrity, immune modulation, and neurotransmission. Their elevated levels in human milk highlight its adaptation to the metabolic needs of the infant, particularly in early life. Studies have shown distinct metabolic responses between breastfed and formula-fed infants [57,58]. Formula feeding leads to higher postprandial levels of amino acids, urea, and nitrogen catabolites, reflecting a greater protein load. In contrast, breastfed infants show higher baseline levels of glutamine, short-chain fatty acids, and ketone bodies, suggesting more balanced energy and amino acid metabolism. These findings support the role of human milk in promoting metabolic homeostasis, while formula may induce more abrupt metabolic shifts.

Some formulas were found to be enriched with tryptophan, an essential amino acid and precursor of serotonin and melatonin. Tryptophan supplementation is often implemented to compensate for its lower natural content in bovine milk proteins, especially casein, and to better replicate the tryptophan-to-large neutral amino acid ratio found in human milk [59]. This enrichment aims to support neurodevelopmental processes and regulate sleep–wake cycles in formula-fed infants. Infant formulas, particularly those designed for the 0–12 months age group, showed a high degree of compositional standardization, forming clusters in multivariate space. However, brand-specific differences were still apparent. For instance, formulations from brands 3 and 7 exhibited higher levels of fucose, maltose, lactulose, and nitrogen-containing compounds, indicative of strategies to mimic human milk’s functional profile or enhance digestibility [12,22,30,60]. Several metabolites, associated with industrial processing, including formate, ascorbate and niacinamide, were elevated in formulas compared to human and animal milk. Formate can be generated during the processing by heating [61], while vitamins like ascorbate and niacinamide, reflect deliberate nutrient fortification, which is performed to compensate for the lack of some bioactive vitamins naturally present in human milk [22,30,62]. Efforts should be made to minimize the formation of undesirable compounds such as formate [63]. Another evidence was the significantly higher concentration of choline in infant formulas. Choline is a critical nutrient for neurodevelopment, synaptic function, and phospholipid biosynthesis, and its presence in higher amounts likely reflects intentional enrichment to meet the needs of infants who are not breastfed [1].

Univariate analysis confirmed the multivariate patterns and highlighted differences in specific metabolite classes. Leucine, hippurate, fumarate, and cis-aconitate were more abundant in animal milks and toddler formulas [31,64,65], whereas glutamine, uridine, and N-acetylated sugars were elevated in human milk and infant formulas [66,67]. These differences likely reflect developmental stage-specific nutritional targets, as well as the influence of processing and ingredient choices [68].

Univariate analyses comparing paired formula products for 0–12 month infants and 1–3 year toddlers revealed specific shifts in metabolite profiles, although these changes were not consistently observed across all brands or product lines. The addition of HMOs such as 3′-FL in toddler formulas may also reflect an effort to simulate the natural increase in this oligosaccharide in later lactation stages. Since our human milk samples span the first five months postpartum, the lower 3′-FL levels observed in infant formulas that include it still fall within the expected physiological range. This emphasizes how formula composition may be informed by detailed knowledge of lactation-stage biochemistry and highlights the need to evaluate not only the presence of specific components, but also their age-appropriate dosage [38].

A limitation of this study is the restricted number and time span of human milk samples, which covered only the early postpartum period.

Because human milk composition undergoes dynamic changes across lactation, future studies including larger cohorts and samples spanning later stages postpartum will be needed to validate and extend our findings.

The greater abundance of glutamine, uridine, and lactose in infant formulas suggests a targeted effort to mirror early-milk nutrition, though differences between brands indicate that this mimicry is not uniform. Elevated creatine, choline, and maltodextrins in toddler formulas align with developmental needs (muscle growth, brain maturation, active metabolism) [1,22,31,69]. However, the increase in formate and maltodextrins highlights manufacturing factors and carbohydrate substitutions that can influence nutritional quality and digestibility [70].

Taken together, our findings highlight the distinct metabolic and functional advantages of human milk, notably its richness in HMOs, free amino acids, and N-acetylated sugars. While goat milk exhibits some similarities, particularly in amino acid composition, it differs in its energy metabolism and protein turnover characteristics. In contrast, cow’s milk is metabolically more divergent, lacking essential oligosaccharides and exhibiting fermentation-related metabolites not aligned with the nutritional needs of early infancy.

## 5. Conclusions

The metabolomic analysis conducted in this study revealed significant differences in the profile of various milk types, with a clear separation between human milk, animal milks, and commercial infant formulas. This metabolomic work based on NMR, demonstrates the power of this untargeted, high-throughput analytical platform in capturing subtle yet functionally relevant variations in complex nutritional matrices. The ability to simultaneously quantify a broad range of compounds, such as amino acids, oligosaccharides, organic acids and sugars, allowed for an in-depth, system-level comparison of nutritional profiles that would be difficult to achieve with conventional analytical methods. Although infant formulas, particularly those for 0–12 months, have significantly improved in recent years, they still represent a simplified model of lactation-derived nutrition. The overall simplification of formula metabolomes, compared to the compositional richness of human milk, supports the conclusion that current formulas, while nutritionally adequate, do not yet replicate the full functional complexity of breast milk. Our analysis also revealed metabolic patterns that appear to reflect age-specific nutritional strategies in formula composition. However, these adjustments are not consistently implemented across all brands. Similarly, while infant formulas are often designed to mimic the composition of human milk, the degree to which they achieve this goal varies considerably. These findings suggest that the final composition of formula products is influenced not only by nutritional goals but also by manufacturing practices. In some cases, industrial standardization may limit the biochemical diversity of formulas, potentially reducing the presence of subtle yet functionally important compounds. Goat’s milk showed partial similarity to human milk in certain metabolic features, but maintained a distinct profile overall, reflecting its species-specific composition. Cow’s milk, on the other hand, was the most metabolically distant, marked by characteristics typical of ruminant digestion and by the absence of several key bioactive components present in human milk.

This study presents several limitations. The number of human milk samples was limited, and they were collected over a relatively narrow lactational window. To capture the full breadth of compositional variability, future studies should include samples from later stages of lactation, including beyond one year, and apply the same principle to animal milks for more meaningful comparisons with formulas consumed during toddlerhood. A similar approach should also be applied to animal milk samples to ensure a more comprehensive comparison. Moreover, our analytical approach is restricted to the analysis of water-soluble metabolome, which is only a limited fraction of the whole complex milk metabolome. Additional analytical strategies could be exploited in further research, to expand the coverage by employing complementary extraction procedures specifically designed to capture the insoluble fraction, including lipidic and protein-associated components. Future research should aim to integrate detailed metabolomic data with clinical outcomes, such as infant growth, gastrointestinal function, neurodevelopmental progress, and microbiota composition. This would allow for a more comprehensive understanding of how specific components of infant formulas influence health in real-world settings. Moreover, longitudinal monitoring of commercial formula products over time, across batches, and in different regulatory contexts would help assess how formulations evolve and how consistently they meet intended nutritional standards. Advances in formulation science, including the incorporation of functional biomimetic ingredients such as synthetic HMOs, bioactive peptides, and postbiotics, should also be explored to improve the functional resemblance of formulas to human milk.

In conclusion, this study offers a comprehensive and up-to-date comparison of the metabolic profiles of human milk, various animal milks, and a broad spectrum of infant formulas. Using NMR-based metabolomics, we reaffirm the distinctive metabolic signature and functional superiority of human milk, highlighting the need for ongoing innovation and transparency in formula development. While notable advancements have been achieved, replicating the full complexity and dynamic nature of breast milk continues to represent a major challenge and a primary objective in pediatric nutrition research.

## Figures and Tables

**Table 1:** Overview of the milk samples included in the study. The table reports the number of batches analyzed for each milk type, brand and year of collection/analysis, divided by age group for infant formulas (0–12 months and 1–3 years). For cow and goat milk, the number of brands and batches is indicated, while for human milk the number of individual donors is reported.

Milk Type/Brand (Year)	n° Batches		
	**0–12 Months**	**1–3 Years**	
Formula Milk—Brand 1 (2019)	5	5	/
Formula Milk—Brand 2 (2019)	5	5	/
Formula Milk—Brand 3 (2019)	4	4	/
Formula Milk—Brand 4 (2019)	5	5	/
Formula Milk—Brand 4 (2025)	4	3	/
Formula Milk—Brand 5 (2019)	5	5	/
Formula Milk—Brand 6 (2025)	3	3	/
Formula Milk—Brand 7 (2025)	4	3	/
Formula Milk—Brand 8 (2025)	4	3	/
Cow milk (conventional, 2025)	/	/	4 brands, 1 batch each
Cow milk (lactose-free, 2025)	/	/	3 batches, same brand
Goat milk (2025)	/	/	4 brands, 1 batch each
Human milk (2019)	/	/	4 individual donors
TOTAL	39	36	15
	90 samples overall		

## Data Availability

Data matrix is available here: https://figshare.com/s/3c4ad5072a1db5205ada, accessed on 16 September 2025.

## Supplementary Materials

The following supporting information can be downloaded at: https://www.mdpi.com/article/10.3390/metabo15090620/s1, Table S1: Table of resonance assignments of detected low-molecular-weight molecules in milk samples. Chemical shift, the proton used for the assignment of detectable components in sample (group) and spectral multiplicity are reported (s, singlet; d, doublet; dd, doublet of doublet; t, triplet; m, multiplet); Figure S1: Biplot of the PCA based on the metabolic profiles of different milk samples (formulas 1–8, human milk 12, cow’s milk 10, lactose-free cow’s milk 9, and goat’s milk 11); Figure S2: Representative ^1^H NMR spectra of the main milk groups (infant formula 0–12 months, toddler formula 1–3 years, cow’s milk lactose-free and conventional, goat’s milk, human milk). Spectra are shown in the 0.8–6.00 (a) and 6.00–10.50 (b) ppm region. Human milk is characterized by strong signals of fucosylated oligosaccharides (2′-FL, 3′-FL, Fuc-α1,3/4-GlcNAc; 5.0–5.5 ppm), N-acetyl carbohydrates (~2.05 ppm), and the amino acids glutamine and glutamate (2.35–2.47 ppm). Infant and toddler formulas display industrial-related markers such as formate (8.46 ppm), elevated choline (3.20 ppm), niacinamide (8.95 ppm), and added carbohydrates including maltodextrins and raffinose (5.3–5.4 ppm region). Cow’s milk shows distinctive orotate (6.19 ppm), mannose (4.9 ppm), and butyrate (1.55 ppm) resonances, while lactose-free cow’s milk presents free glucose (3.26 ppm) and galactose (5.27 ppm) instead of intact lactose (3.33 ppm). Goat’s milk is distinguished by higher creatine/carnitine signals (3.0–3.2 ppm), succinate (2.39 ppm), uridine (7.86 ppm), and inosine (8.24 ppm). Together, these spectral regions highlight the unique metabolic signatures of each milk group, in agreement with the analyses presented in the main text; Figure S3: Heatmap of the z-score values of metabolites identified by NMR in the different milk samples analyzed (in this case, the formulas 1–3 years, groups 1–8, group 12, lactose-free cow’s milk, group 9, cow’s milk 10, and goat’s milk, group 11); Table S2: Mean ± Standard deviations of 0–12 months formula milk (1–8), HD cow milk (9), cow milk (10), goat milk (11) and human milk (12); Table S3: Table of *p*-values for amino acids; Figure S4: Boxplot of the levels of amino acids performed to assess overall differences among groups 1 to 8 or A to I (0–12 months new born formulas), 9 or LF (lactose free) HD cow’s milk, 10 or Bt (Bos taurus) conventional cow’s milk, 11 or Ch (Capra hircus) goat’s milk, 12 of H (human milk); Table S4. Table of *p*-values for organic acids; Figure S5: Boxplot of the levels of organic acids performed to assess overall differences among groups 1 to 8 or A to I (0–12 months infant formulas), 9 or LF (lactose free) HD cow’s milk, 10 or Bt (Bos taurus) conventional cow’s milk, 11 or Ch (Capra hircus) goat’s milk, 12 of H (human milk); Table S5: Table of *p*-values for amines and derivatives; Figure S6: Boxplot of the levels of amines and derivatives performed to assess overall differences among groups 1 to 8 or A to I (0–12 months infant formulas), 9 or LF (lactose free) HD cow’s milk, 10 or Bt (Bos taurus) conventional cow’s milk, 11 or Ch (Capra hircus) goat’s milk, 12 of H (human milk); Table S6: Table of *p*-values for vitamins; Figure S7: Boxplot of the levels of vitamins performed to assess overall differences among groups 1 to 8 or A to I (0–12 months infant formulas), 9 or LF (lactose free) HD cow’s milk, 10 or Bt (Bos taurus) conventional cow’s milk, 11 or Ch (Capra hircus) goat’s milk, 12 of H (human milk); Table S7: Table of *p*-values for carbohydrates and sugars; Figure S8: Boxplot of the levels of carbohydrates and sugars performed to assess overall differences among groups 1 to 8 or A to I (0–12 months infant formulas), 9 or LF (lactose free) HD cow’s milk, 10 or Bt (Bos taurus) conventional cow’s milk, 11 or Ch (Capra hircus) goat’s milk, 12 of H (human milk); Table S8: Table of *p*-values for energetic compounds, short chain fatty acids (SCFA), nucleotides and derivatives and other compounds; Figure S9: Boxplot of the levels of energetic compounds (acetyl-carnitine, carnitine and glycerophosphocholine); Table S9: Metabolites’ mean ± standard deviations of 0–12 months and 1–3 years formula milk (brands from 1 to 8), lactose free cow milk (9), cow milk (10), goat milk (11) and human milk (12); Figure S10: Dumbbell plot of the amino acids, organic acids, amine and derivatives and vitamins; Figure S11: Dumbbell plot of the carbohydrates and sugars, energetic compounds, short chain fatty acids, nucleotides and derivatives and other compounds; Figure S12: The figure displays the signals of isoleucine, leucine, and valine for Brand 7, formulation 1–3y; Figure S13: The figure displays the characteristic tryptophan (Trp) signals for two specific formulations: Brand 7 (1–3y) and brand 4 (0–12m) from the previous sample collection.

## Abbreviations

The following abbreviations are used in this manuscript:

2′-FL	2′-Fucosyllactose
3′-FL	3′-Fucosyllactose
AcCar	Acetyl-carnitine
Ace	Acetone
Ala	Alanine
Ara	Arabinose
Asc	Ascorbate
Bu	Butyrate
Car	Carnitine
Cho	Choline
Cit	Citrate
c-Aco	Cis-aconitate
Cr	Creatine
Crn	Creatinine
DMA	Dimethylamine
DMSO_2_	Dimethylsulfone
d	Doublet
EtOH	Ethanol
FDR	False Discovery Rate
FDR *p*-value	Valore di *p* corretto per FDR
Fuc	Fucose
Fuc-α1,3-GlcNAc	Fucosyl-α-1,3-N-acetylglucosamine
Fuc-α1,4-GlcNAc	Fucosyl-α-1,4-N-acetylglucosamine
FM	Formula Milk
For	Formate
Fum	Fumarate
Gal	Galactose
Glc	Glucose
Gln	Glutamine
Glu	Glutamate
GPC	Glycerophosphocholine
Hip	Hippurate
His	Histidine
HM	Human Milk
HMO	Human Milk Oligosaccharides
ILE	Isoleucine
Ino	Inosine
Lac	Lactose
Lcl	Lactulose
LD	Lactose-free
Leu	Leucine
log2FC	Log_2_ Fold Change
MA	Methylamine
Man	Mannose
MDX	Maltodextrin
m	Multiplet
NAc-Carb	N-acetyl carbohydrates
NAM	Niacinamide
NMR	Nuclear Magnetic Resonance
Oro	Orotate
PC	Principal Component
PCA	Principal Component Analysis
PCr	Phosphocreatine
Phe	Phenylalanine
q	Quartet
Raf	Raffinose
RCF	Relative Centrifugal Force
s	Singlet
SCFA	Short Chain Fatty Acids
Suc	Sucrose
Suc	Succinate
t	Triplet
TMA	Trimethylamine
TSP	Trimethylsilylpropionic acid
Trp	Tryptophan
Tyr	Tyrosine
UDP-Gal	UDP-galactose
UDP-Glc	UDP-glucose
Urd	Uridine
Val	Valine
