# Supplementary material for: NMR-Based Metabolomic Profiling Highlights Functional Nutritional Gaps Between Human Milk, Infant Formulas, and Animal Milks
Source: Metabolites. 2025 Sep 18;15(9):620. doi: 10.3390/metabo15090620 (PMC12471977; doi:10.3390/metabo15090620)
Supplement: Supplementary file 1 [file metabolites-15-00620-s001.zip › metabolites-3839650-supplementary.pdf]

Table S1. Table of resonance assignments of detected low-molecular-weight molecules in milk samples. Chemical shift, the proton used for the assignment of detectable components in sample (group) and spectral multiplicity are reported (s, singlet; d, doublet; dd, doublet of doublet; t,triplet; m, multiplet).

Metabolite	Chemical shift (ppm)	Group /Multiplicity
Leucine	0.96	CH_3, t
Isoleucine	1.01	CH_3, d
Valine	1.03	CH_3, d
Ethanol	1.18	CH_3, t
Lactate	1.33	CH_3, d
Alanine	1.48	CH_3, d
Butyrate	1.55	CH_2, m
Acetate	1.91	CH_3, s
N-acetyl carbohydrates	2.05	CH_3, s
Acetone	2.22	CH_3, s
Glutamate	2.35	CH_2, m
Succinate	2.39	$2\times CH_2$, s
2-oxoglutarate	2.42	CH_2, t
Glutamine	2.47	CH_2, m
Acetyl-carnitine	2.49	CH_2, q
Citrate	2.53	CH_2, d
Methylamine	2.6	CH_3, s
Dimethylamine	2.72	$2\times CH_3$, s
Trimethylamine	2.9	$3\times CH_3$, s
Creatine	3.04	CH_3, s
Creatinine + Phosphocreatine	3.05	CH_3, s
Dimethyl sulfone	3.15	$2\times CH_3$, s
Choline	3.2	CH_3, s
Carnitine	3.22	$3\times CH_3$, s
Glycerophosphocholine	3.23	$3\times CH_3$, s
Glucose	3.26	CH, dd
Lactose	3.33	CH, t
Sucrose	4.05	CH, t
Lactulose	4.14	CH, dd
Ascorbate	4.5	CH, d
Arabinose	4.54	CH, d
Fucose	4.55	CH, d
Mannose	4.89	CH, d
Raffinose	4.99	CH, d
Fucosyl-α-1,4-N-acetylglucosamine	5.04	CH, d
Fucosyl-α-1,3-N-acetylglucosamine	5.16	CH, d
Galactose	5.27	CH, d
2’-Fucosyllactose	5.3	CH_3, m
Maltodextrin	5.4	CH, m
3’-Fucosyllactose	5.46	CH_3, m

UDP-glucose	5.59	CH, q
UDP-galactose	5.65	CH, q
cis-Aconitate	5.74	CH, s
Orotate	6.19	CH, s
Fumarate	6.51	2×CH, s
Tyrosine	6.91	2×CH, d
Histidine	7.08	CH, s
Phenylalanine	7.45	2×CH, m
Hippurate	7.64	2×CH, m
Tryptophan	7.75	CH, d
Uridine	7.86	CH, d
Inosine	8.24	CH, s
Formate	8.46	CH, s
Niacinamide	8.95	CH, dd

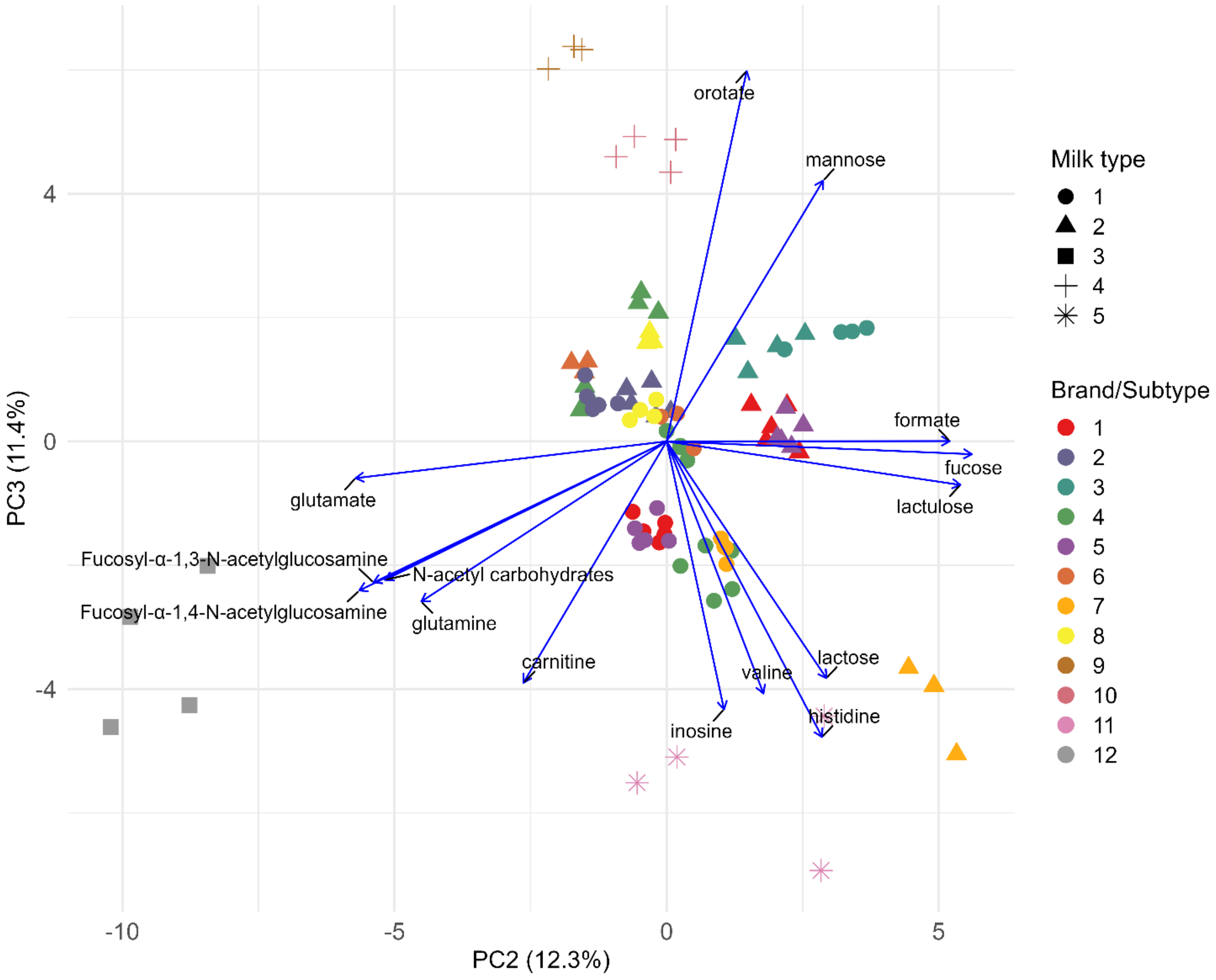

Figure S1. Biplot of the PCA based on the metabolic profiles of different milk samples (formulas 1–8, human milk 12, cow's milk 10, lactose-free cow's milk 9, and goat's milk 11). The symbols and colors distinguish the types of milk analyzed. The circle symbol corresponds to milk formulas for newborns 0–6 months, the triangle corresponds to follow-up formulas for 1–3 years, the square represents human breast milk samples, the cross represents cow's milk, and the asterisk represents goat's milk. The blue arrows represent the loadings of metabolites measured by NMR, indicating the direction and contribution of each compound to the variance explained by PC2 and PC3.

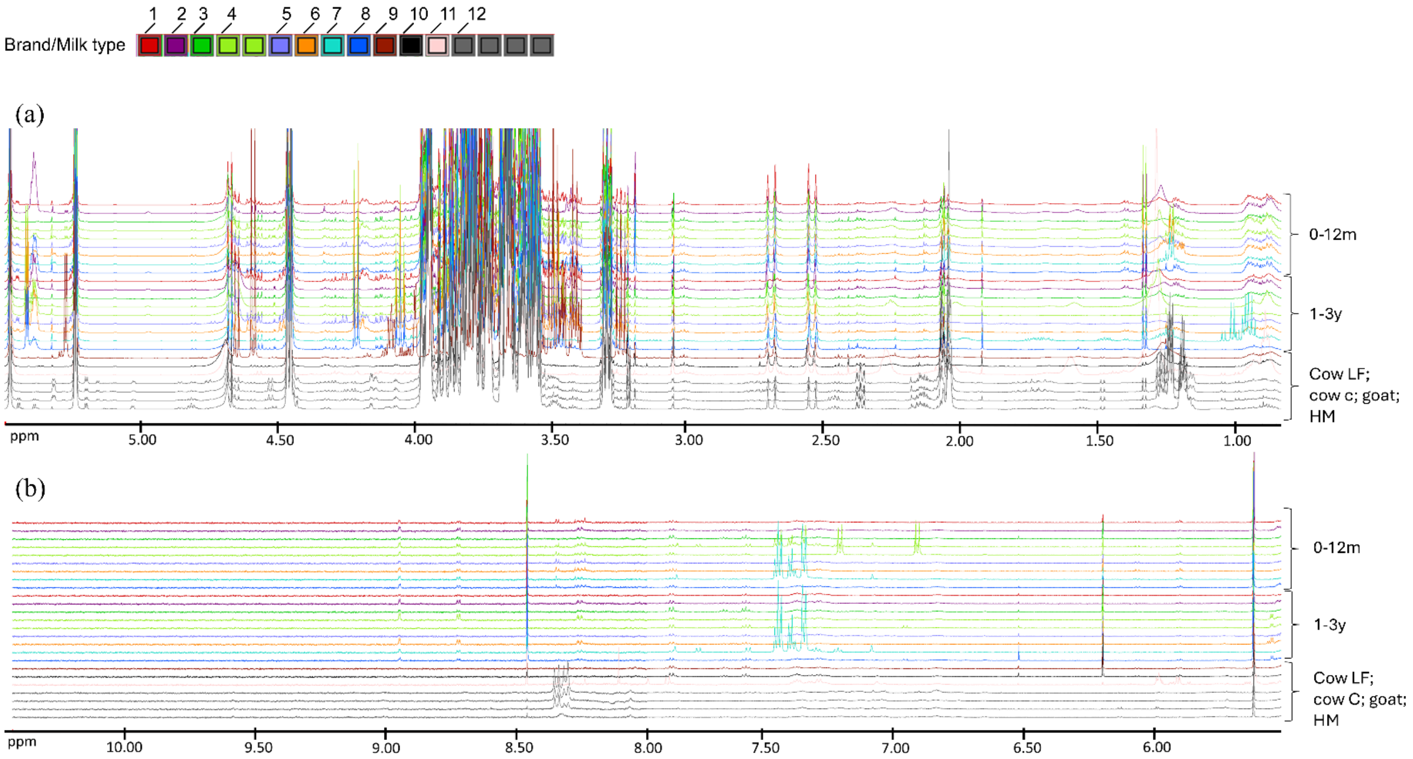

Figure S2. Representative ^{1}H NMR spectra of the main milk groups (infant formula 0-12 months, toddler formula 1–3 years, cow's milk lactose-free and conventional, goat's milk, human milk).
Spectra are shown in the 0.8-6.00 (a) and 6.00-10.50 (b) ppm region. Human milk is characterized by strong signals of fucosylated oligosaccharides (2'-FL, 3'-FL, Fuc-α1,3/4-GlcNAc; 5.0-5.5 ppm), N-acetyl carbohydrates (~2.05 ppm), and the amino acids glutamine and glutamate (2.35-2.47 ppm). Infant and toddler formulas display industrial-related markers such as formate (8.46 ppm), elevated choline (3.20 ppm), niacinamide (8.95 ppm), and added carbohydrates including maltodextrins and raffinose (5.3-5.4 ppm region). Cow's milk shows distinctive orotate (6.19 ppm), mannose (4.9 ppm), and butyrate (1.55 ppm) resonances, while lactose-free cow's milk presents free glucose (3.26 ppm) and galactose (5.27 ppm) instead of intact lactose (3.33 ppm). Goat's milk is distinguished by higher creatine/carnitine signals (3.0-3.2 ppm), succinate (2.39 ppm), uridine (7.86 ppm), and inosine (8.24 ppm). Together, these spectral regions highlight the unique metabolic signatures of each milk group, in agreement with the analyses presented in the main text.

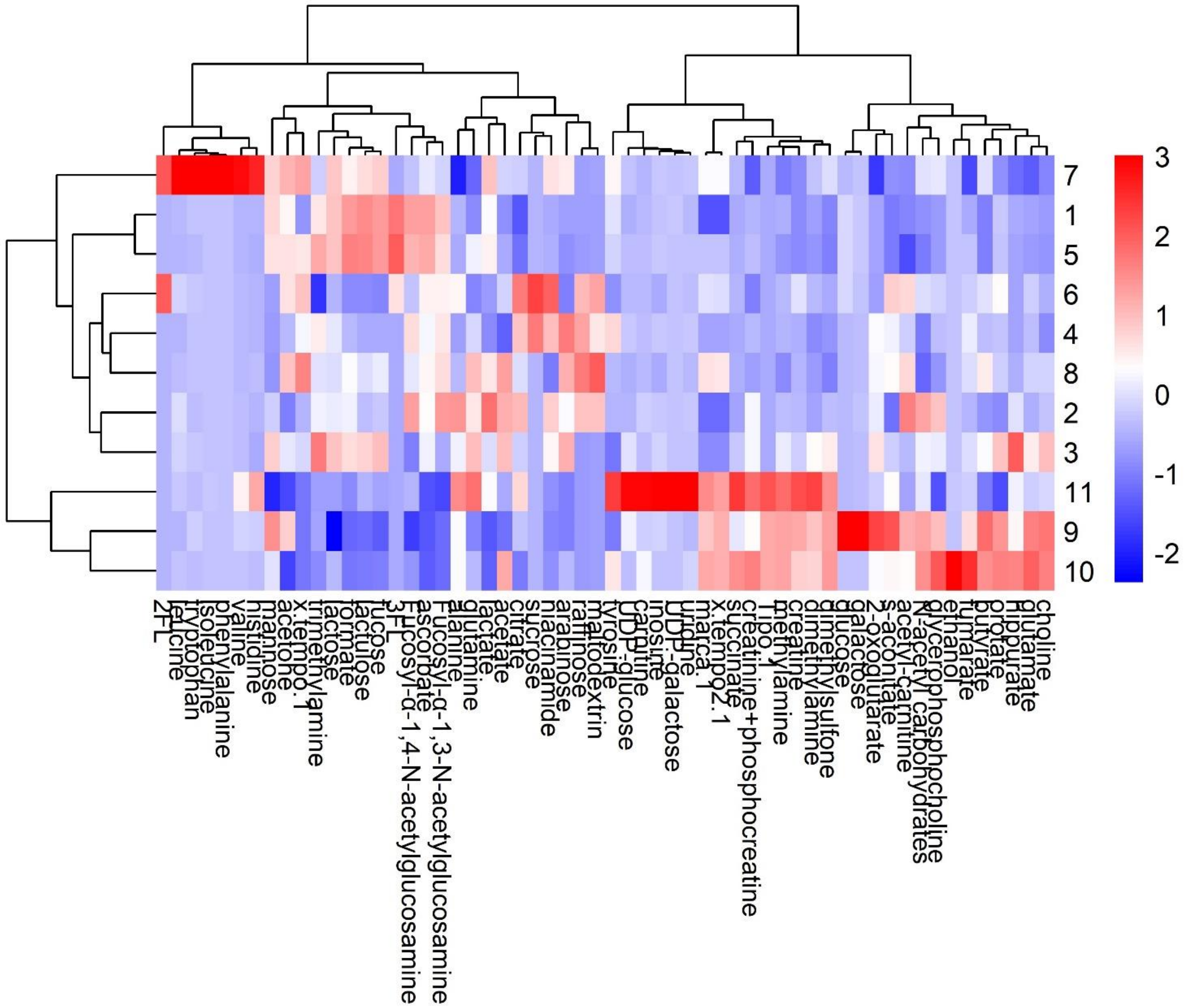

Figure S3. Heatmap of the z-score values of metabolites identified by NMR in the different milk samples analyzed (in this case, the formulas 1–3 years, groups 1–8, group 12, lactose-free cow's milk, group 9, cow's milk 10, and goat's milk, group 11). The rows represent the milk categories (1–11), while the columns correspond to the metabolites. The color scale varies from blue (negative z-score values) to red (positive z-scores), representing values below or above the global mean of the respective metabolite. The distance between groups (rows) and metabolites (columns) was calculated using Euclidean distance, and hierarchical clustering was performed with the Ward.D2 agglomeration method to generate dendrograms representing the similarity of the average profiles of the brands.

Table S2. Mean ± Standard deviations of 0-12 months formula milk (1-8), HD cow milk (9), cow milk (10), goat milk (11) and human milk (12).

Amino acids	1	2	3	4	5	6	7	8	9	10	11	12
alanine	990.9±47.8	1162.9±92.8	915.6±73.7	1243.2±204.4	1008.4±43.5	1339.6±27.1	817.6±25.2	1134.1±47.7	803.6±123	803.9±59.8	1219±263.1	1142.5±194.4
glutamine	72.2±20.9	66.5±14.7	89.8±24.3	83.5±16.9	59.6±20.3	116.5±11.5	49.8±26.4	108.7±2.9	23.6±21.3	39.6±30.1	105.4±56.8	452.4±254.2
glutamate	73.3±10.6	113.7±9.4	357.7±10.3	161.2±28.5	90.3±12.1	156.6±4.5	71.1±4.6	87.7±12.7	532.5±26.4	581.3±16.3	261.8±19.5	2712.6±763.5
histidine	48.3±36.1	38.1±12.1	46.4±18.6	164.5±117	68.7±43.3	53.5±47.7	336.2±29.8	46.6±36.9	72.7±36.7	52±12.8	270.4±118.9	26±32.6

isoleucine	7.9±17.6	27.1±28	0±0	7.6±22.9	4.8±10.8	0±0	10.9±9.7	13.7±19.9	3.6±6.2	0±0	33±66	0±0
leucine	163.2±29.1	156.2±16.9	151.9±18.8	207.7±37.7	161.4±35.4	233.8±11.6	149.6±21.1	141.2±22.5	0±0	46±53.3	74.9±52	0±0
phenylalanine	23±19.2	4.6±8.3	1.9±3.8	1173.5±1101.3	27.4±34.6	24.6±25.7	5894±78.7	8±16.1	0±0	2.9±5.8	16.7±19.3	73.6±58
tryptophan	10.4±10.1	15.1±19.8	12.1±7	167.1±134.9	21.3±15.1	19±26.2	21.1±14.8	12.4±17.8	30.9±15	12.5±10.8	14.7±17.9	12.8±8.8
tyrosine	25.7±18.5	25.1±16.9	9.2±10	1690.3±1023.3	17.7±26.4	13.2±13.3	45.8±15.7	20.2±35.9	7.1±8.6	27.9±22	50.1±19.6	31.2±46.7
valine	137.3±36.8	95.2±39.6	108.3±25.3	188.3±70.9	124.6±30.3	163.4±5.2	72.4±43.8	55.6±28.7	269.6±106.7	155±23.3	654.1±161.8	419±165.7
**Organic acids**												
2-oxoglutarate	25.7±5.6	42.1±13.5	85.7±9.1	56.2±33.2	22.6±8.4	87.6±10.3	17.4±4.1	87.3±10.6	109.8±13	57.8±12.6	44.8±45	26.7±28.8
cis-aconitate	14.7±17.6	21.6±25.2	32.6±26.4	32±27.1	31.2±29.9	44.3±29.8	31.4±27.4	3.6±6.1	101.5±16.6	60.7±33.1	60.4±49.7	78±41.2
citrate	15663.6±662.9	8748.2±494.1	11634.7±510.2	10780.2±2944.2	15644.6±458.2	10599.8±304	13332.2±495.4	11935.9±627.9	10963±208.7	8167.4±3431.4	14687.5±7762.4	4838.5±3169.5
formate	275.2±72.4	289.5±63.2	728.2±40.2	284.4±56	249.4±60.3	449.3±6	310.1±75.7	284.3±46	44.3±6.3	73.2±10.2	111±106.4	23±8
fumarate	22.7±9	17.1±6.1	43.3±12.1	18.4±12.3	28.4±11.6	26.8±11.6	12.8±9.8	26.5±9.7	55.2±18.9	87.4±7.7	40.8±10.6	10.6±5.3
hippurate	41.4±30.3	101.1±20.4	217.2±40.1	59.6±36	59.7±28.8	51.7±22.4	47.7±3.1	70±27.3	158.9±20	226.1±67.6	148.6±45.9	23±17
lactate	1489.6±95.9	5887.4±837.9	2341.7±241.1	5990.3±4966.6	1460.1±82	7451.5±74.2	1695.8±323.6	3729.3±2192.8	464.7±34.8	596.6±241.9	3364.2±5067.1	947.7±506.3
succinate	136.5±5.8	115.2±7.2	359.4±53.8	147.7±52.8	124.9±19.5	195±6.5	163±27.4	176.9±30	357.8±75.2	800.6±268.7	1075.8±340.7	89.1±25.6
**Amines and derivatives**												
choline	18747.7±375	32719.4±760.3	7212.7±773	14418.1±9509.5	18917.1±1134.8	18833.3±80.8	25655.6±429.4	39785.4±744	8465.4±773.2	8297.5±1707.5	5227.6±3124.1	4063.5±1938.1
creatine	246.4±111.8	778.8±170.2	832.4±85.6	689.4±335.7	267.8±76.7	838±8.2	293.4±99.8	374.3±54.9	3043.7±290.2	2587.1±422.2	4264.6±420.2	70.5±99.4
creatinine+phosphocreatine	997±80.5	1831±153.4	2852.5±67.4	2468.3±363.5	1014.3±69.3	2011.7±18.6	1007.5±97.1	1215.1±77.8	2303.3±271.8	3089.1±478.7	3227.2±779.4	1475.3±94.7
dimethylamine	60.6±11.5	97±4.9	286.8±55.8	88.8±22.4	53.3±4.8	155.9±9.4	204.2±36.2	88.3±12.2	282.8±8.6	269±69.6	427.8±365.2	141±91.3
methylamine	13.1±9.2	10.9±11.4	33.2±3.2	8.5±8.6	15.7±6.3	22.6±2.2	5±3.2	9.4±4.8	50.5±5	51.2±16.2	63±14.3	9.1±7
trimethylamine	14.1±7.3	32.3±10	155±49.8	19.4±17.3	8.5±7.7	61.5±5.4	25.7±6.4	25.2±7	34.2±12.8	29.3±13.1	32.7±31.7	3.9±7.1
**Vitamins**												
ascorbate	1987.9±85.5	2035.4±410.4	1351.7±221	2732±567.2	1995.1±76.4	2220±124.7	2320.6±282.1	2173.2±369.8	664.8±35.7	763.1±496.3	600.6±44.4	2006.6±369.4
niacinamide	59.2±7.1	107.2±13.1	56.4±11.8	93.9±16.9	68.1±19.4	78.3±10.5	75.6±6.3	82.8±5	12.7±13.8	18.1±11.2	18.1±8.8	8.3±6.6
**Carbohydrates and sugars**												
2'-fucosyllactose	0±0	0±0	0±0	1329±1576.8	0±0	1797±551.6	2518±95	0±0	0±0	0±0	0±0	3923±3015
3'-fucosyllactose	3721.8±237	0±0	0±0	2444±729	3854±170	0±0	0±0	3804±143	0±0	0±0	0±0	1931±2216
arabinose	20.7±29.5	35.6±36.4	159.5±29.5	2.8±5.9	17.6±10.2	132.6±229.7	0±0	0±0	0±0	44.7±48.5	0±0	124.4±248.9
fucose	613.3±96.7	909.6±91	2700.2±83.8	1154.1±451.6	581.7±131.6	843.1±8.2	1440.9±107.4	741.9±75.1	59.1±51.3	432.6±147.3	858.6±714	32±40.4
Fucosyl-α-1,3-N-acetylglucosamine	295.3±13.3	506±28.9	273.9±38.6	335.3±19.8	298.1±19.8	344±58.9	244.3±46.5	332.2±43.9	184.7±40.4	186.7±84	144.2±12.9	2284.1±1488.8
Fucosyl-α-1,4-N-acetylglucosamine	225.4±7.4	303±43.7	132.1±89.6	235.8±41.7	228.3±13.4	237±105.6	135.7±49.7	233.3±109	20.6±35.6	134.2±97.8	169.1±53.3	4168.5±1523.7
galactose	2297.9±159.7	4251±425.7	1955.9±80.3	4383.5±669.8	2260.5±167.2	4489±80.5	2130.4±43.4	2499.3±172.8	104145.3±439.1	2967.4±4109.5	700.7±119.5	1142.5±294.9
glucose	11355±1007.8	0±0	0±0	2624.2±2849	11317±361.8	0±0	0±0	9318.8±324.7	167469.9±836.6	4181.1±4833.1	0±0	0±0
lactose	289695.3±3864	208143.4±10272.5	331919.9±23037.9	303521.9±13587	291475.8±3928.4	291497.5±315.4	286103.7±5354.5	177372.5±4839.6	0±0	196091.3±11157.7	169632.7±7726	300481.5±6423.9
lactulose	828.7±104.9	718±123.2	2782.5±77.8	1232±550.5	823.9±149.5	584.9±12.9	1426.5±131.6	956.7±53	10.8±18.8	234.9±163.8	1022.7±614.4	0±0
maltodextrin	1148±135.1	134391.7±8443.6	0±0	5989.3±6932.4	1124.5±130.5	12700±32	2119.7±206.8	89846.4±5302.2	0±0	0±0	0±0	3109.6±2362.7

mannose	28.7±5.5	53±8.6	62.4±7.6	59±16.2	26.2±11	59.3±9.8	47±6.8	33.7±4.2	78.8±22.8	50.2±14.2	10.9±12.5	26.8±10.7
N-acetyl carbohydrates	10746.9±255.2	16140.4±1025.5	14834.2±950.5	18319.3±2815.1	10980.6±411.1	24784.1±320.3	17182.5±259.6	11073.3±490.5	16933.7±765.8	17627.9±514.2	13523.7±3521.6	79389.8±38608.9
raffinose	3.8±5.9	4248.2±438.5	0±0	202.5±232.6	5.9±13.3	309±30.2	16.2±17.2	2607.6±170.8	0±0	0±0	0±0	4.2±8.5
sucrose	0±0	0±0	0±0	0±0	0±0	0±0	0±0	0±0	0±0	0±0	0±0	0±0
UDP-galactose	3.7±8.3	0.8±1.8	0±0	0.1±0.2	3.3±7.3	0±0	4.1±6.1	0±0	0±0	4.8±8.9	415±132.2	0±0
UDP-glucose	1.8±4	5.3±5.3	4.9±6	10.5±11.3	5.4±5.4	9.3±5	8.4±11.9	3.8±7.3	17±21.7	5.1±6.1	80.4±43.1	7.5±3.4
**Energetic compounds**												
acetyl-carnitine	4.2±3	21.6±4.3	8.4±5.2	20.3±6.1	4±2.3	16.3±5.3	8.9±3.8	12.8±3.2	24.1±4.4	17.5±3.5	9.7±7.5	18.9±13.6
carnitine	502.2±379.8	1612±153.6	1294.3±73.2	1949±920.3	350.2±133.3	1069.9±33.3	536.2±99.7	338.9±3	1403±237.3	2666.9±227.1	10172±846.7	12280±4176.3
glycerophosphocholine	2022.9±298.5	11180.1±873.9	7482.4±904.7	12900.4±991.1	2062.5±271.6	14511.5±349.4	5391.4±320.2	3553.5±313.2	9649.2±1024.2	12341.9±1481.1	3457.6±2396.4	6243.7±2131.3
**Short chain fatty acids (SCFA)**												
acetate	337.8±35	711.9±115.1	1017.7±32.4	859.6±216.1	326.1±40	869.4±12.6	466.7±78	552.5±66.7	436.5±83.6	826.4±331	534.1±111.3	108±115.5
butyrate	2.4±5.3	11.7±16.7	59.7±48.1	12.6±19	2.9±6.5	23.2±28.3	21.5±23.7	10.5±10.2	113.1±14.1	99.1±12.8	0±0	127±170.4
**Nucleotides and derivatives**												
inosine	32.2±6.8	9.4±8.8	5.9±5	35.8±6.6	34.7±7.1	23.9±4.6	6.4±6.1	15.5±7.6	10.6±8.7	6.8±5.5	73±21	2.9±3.4
uridine	27.8±6.5	9.6±7.6	17.8±13.2	35.3±10.3	29.7±10.6	46.5±23	28±5.2	17.9±5.4	9.1±11.8	24.9±5.9	234.7±21.2	16±26.5
orotate	217.3±20.9	211.3±16.2	476.3±43.1	233.2±25.3	216.4±56.4	321.5±19.8	295.2±61.6	247.3±21.3	798±43.8	844.2±59.4	142.3±46.1	22.7±5.3

Table S3. Table of p-values for amino acids. For each metabolite, a Kruskal-Wallis test was performed to assess overall differences among groups 1 to 8 (0-12 months infant formulas), 9 HD cow's milk, 10 conventional cow's milk, 11 goat's milk, 12 human milk. When significant, pairwise comparisons were conducted using Dunn's post-hoc test, with p-values adjusted using the False Discovery Rate (FDR) method. The Cfr column indicates the pair of groups being compared (e.g., 1–2 denotes the comparison between group 1 and group 2). FDR-adjusted p-values < 0.05 are considered statistically significant and are highlighted in red.

Cfr	alanine	glutamine	glutamate	histidine	isoleucine	leucine	phenylalanine	tryptophan	tyrosine	valine
1-2	0.196	0.708	0.286	0.776	0.323	0.806	0.471	0.997	0.996	0.406
1-3	0.750	0.559	0.007	0.941	0.796	0.797	0.441	0.997	0.657	0.587
2-3	0.099	0.366	0.115	0.873	0.113	0.946	0.862	0.997	0.657	0.809
1-4	0.094	0.617	0.035	0.310	0.908	0.299	0.537	0.454	0.032	0.536
2-4	0.920	0.395	0.481	0.108	0.113	0.170	0.097	0.454	0.032	0.108
3-4	0.041	0.827	0.311	0.308	0.845	0.174	0.092	0.480	0.008	0.239
1-5	0.857	0.617	0.606	0.759	1.000	0.842	0.883	0.971	0.770	0.733
2-5	0.318	0.900	0.580	0.483	0.323	0.946	0.537	0.954	0.770	0.624
3-5	0.582	0.341	0.028	0.759	0.796	0.912	0.471	0.997	0.909	0.826
4-5	0.190	0.341	0.151	0.727	0.920	0.179	0.471	0.887	0.008	0.313
1-6	0.074	0.196	0.115	0.893	0.796	0.288	0.986	0.997	0.770	0.587
2-6	0.582	0.117	0.583	0.759	0.172	0.174	0.537	0.997	0.770	0.215
3-6	0.040	0.497	0.463	0.876	1.000	0.174	0.471	0.997	0.943	0.318
4-6	0.644	0.341	1.000	0.575	0.856	0.827	0.599	0.660	0.023	0.916
5-6	0.130	0.097	0.286	0.876	0.796	0.186	0.906	0.997	0.996	0.405
1-7	0.311	0.341	0.804	0.053	0.796	0.574	0.092	0.997	0.637	0.217
2-7	0.025	0.506	0.212	0.027	0.796	0.805	0.010	0.997	0.637	0.627
3-7	0.582	0.125	0.007	0.053	0.556	0.842	0.010	0.997	0.267	0.507

4-7	0.012	0.120	0.028	0.315	0.658	0.105	0.288	0.660	0.502	0.043
5-7	0.196	0.576	0.516	0.123	0.796	0.787	0.092	0.997	0.319	0.369
6-7	0.012	0.030	0.090	0.123	0.610	0.123	0.150	0.997	0.368	0.106
1-8	0.263	0.215	0.652	0.941	0.796	0.494	0.599	0.997	0.770	0.152
2-8	0.980	0.120	0.580	0.873	0.796	0.787	0.862	0.997	0.770	0.536
3-8	0.142	0.552	0.033	0.991	0.556	0.805	0.798	0.997	0.911	0.406
4-8	0.857	0.357	0.168	0.308	0.611	0.104	0.237	0.454	0.010	0.025
5-8	0.389	0.099	0.969	0.759	0.796	0.719	0.705	0.954	0.996	0.272
6-8	0.582	0.889	0.286	0.876	0.583	0.105	0.670	0.997	0.996	0.073
7-8	0.034	0.030	0.570	0.053	1.000	0.946	0.035	0.997	0.344	0.866
1-9	0.350	0.196	0.007	0.759	1.000	0.073	0.380	0.660	0.637	0.260
2-9	0.034	0.345	0.090	0.556	0.556	0.105	0.777	0.660	0.637	0.055
3-9	0.610	0.092	0.804	0.759	0.796	0.124	0.862	0.741	0.964	0.108
4-9	0.019	0.087	0.241	0.759	0.856	0.002	0.092	0.997	0.008	0.475
5-9	0.245	0.395	0.026	0.964	1.000	0.104	0.441	0.997	0.902	0.147
6-9	0.019	0.024	0.342	0.876	0.796	0.006	0.441	0.887	0.902	0.591
7-9	0.989	0.708	0.007	0.225	0.796	0.176	0.010	0.954	0.267	0.024
8-9	0.044	0.024	0.028	0.759	0.796	0.196	0.647	0.660	0.902	0.018
1-10	0.306	0.341	0.002	0.927	0.796	0.082	0.441	0.997	0.996	0.667
2-10	0.025	0.506	0.030	0.759	0.113	0.123	0.868	0.997	0.996	0.239
3-10	0.582	0.125	0.606	0.876	1.000	0.164	0.986	1.000	0.657	0.371
4-10	0.012	0.120	0.100	0.464	0.845	0.002	0.092	0.480	0.068	0.897
5-10	0.196	0.576	0.007	0.873	0.796	0.105	0.471	0.997	0.770	0.469
6-10	0.012	0.030	0.212	0.964	1.000	0.006	0.471	0.997	0.770	0.866
7-10	0.989	1.000	0.002	0.080	0.556	0.222	0.010	0.997	0.637	0.108
8-10	0.034	0.030	0.008	0.876	0.556	0.268	0.803	0.997	0.770	0.076
9-10	0.989	0.708	0.804	0.873	0.796	0.847	0.862	0.741	0.637	0.475
1-11	0.221	0.616	0.018	0.080	0.908	0.105	0.803	0.997	0.637	0.054
2-11	0.989	0.414	0.216	0.039	0.556	0.166	0.705	0.997	0.637	0.005
3-11	0.119	0.925	0.778	0.080	0.796	0.186	0.599	0.997	0.280	0.018
4-11	0.926	0.900	0.522	0.483	0.796	0.003	0.441	0.480	0.452	0.119
5-11	0.343	0.357	0.065	0.225	0.908	0.150	0.862	0.997	0.344	0.024
6-11	0.610	0.433	0.606	0.199	0.796	0.010	0.853	0.997	0.412	0.260
7-11	0.031	0.150	0.013	0.876	0.845	0.299	0.088	0.997	0.996	0.003
8-11	0.980	0.506	0.073	0.080	0.805	0.355	0.857	0.997	0.368	0.002
9-11	0.040	0.099	0.606	0.317	1.000	0.797	0.502	0.723	0.280	0.587
10-11	0.031	0.150	0.463	0.123	0.796	0.898	0.599	0.997	0.657	0.160
1-12	0.350	0.053	0.001	0.759	0.796	0.042	0.471	0.997	0.902	0.108
2-12	0.857	0.024	0.013	0.893	0.113	0.091	0.106	0.997	0.902	0.018
3-12	0.196	0.196	0.463	0.759	1.000	0.105	0.092	0.997	0.800	0.041
4-12	0.699	0.092	0.041	0.085	0.845	0.001	0.855	0.480	0.025	0.239
5-12	0.538	0.024	0.003	0.402	0.796	0.082	0.441	0.997	0.902	0.053
6-12	0.454	0.617	0.115	0.690	1.000	0.003	0.537	0.997	0.902	0.405
7-12	0.044	0.005	0.001	0.027	0.556	0.166	0.471	0.997	0.487	0.005
8-12	0.920	0.506	0.004	0.759	0.556	0.174	0.230	0.997	0.902	0.004
9-12	0.072	0.005	0.606	0.464	0.796	1.000	0.092	0.741	0.770	0.773
10-12	0.044	0.005	0.778	0.690	1.000	0.842	0.092	0.997	0.902	0.272

11-12	0.857	0.168	0.286	0.039	0.796	0.787	0.391	0.997	0.525	0.773

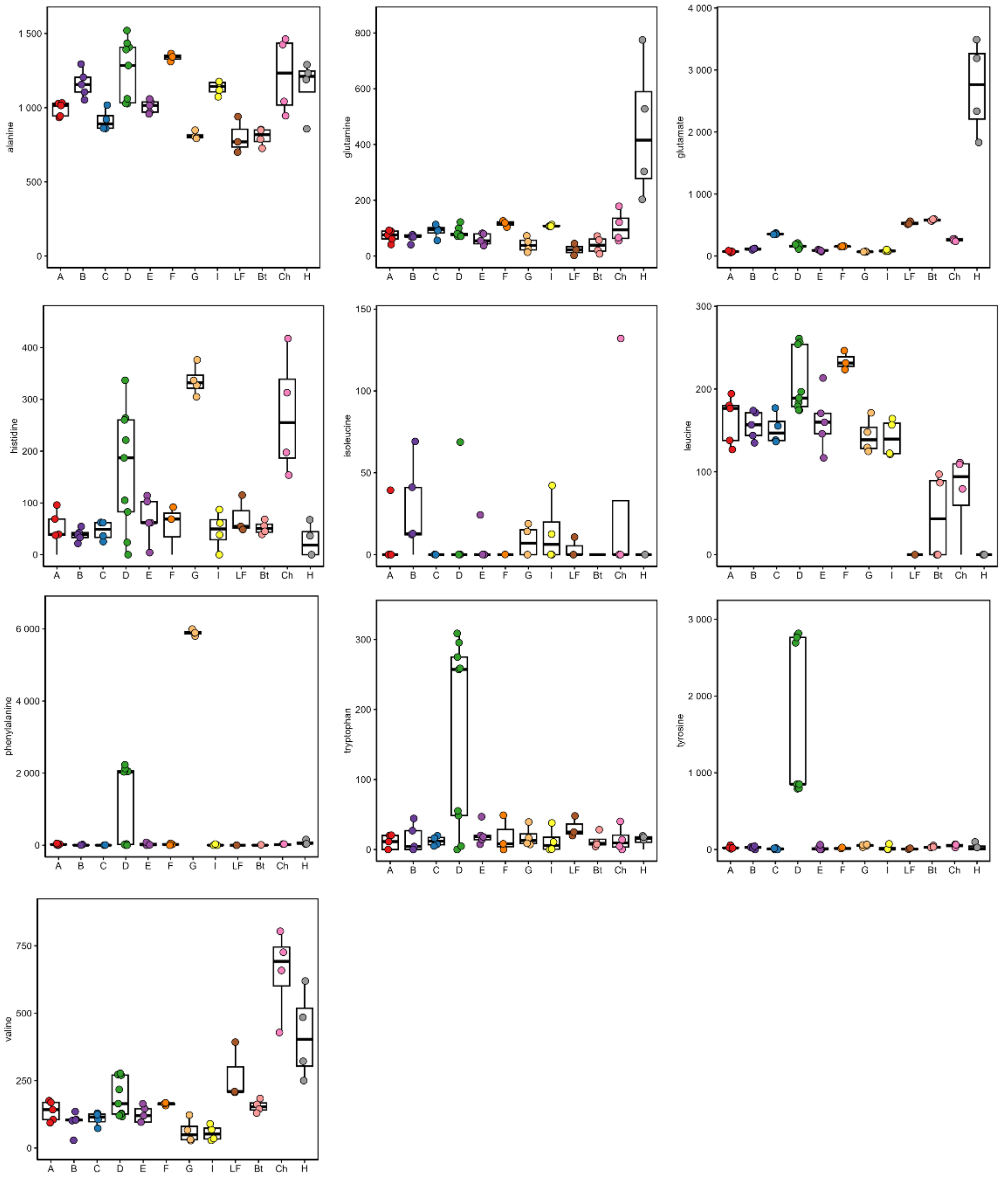

Figure S4. Boxplot of the levels of amino acids performed to assess overall differences among groups 1 to 8 or A to I (0-12 months new born formulas), 9 or LF (lactose free) HD cow's milk, 10 or Bt (Bos taurus) conventional cow's milk, 11 or Ch (Capra hircus) goat's milk, 12 of H (human milk).

Table S4. Table of p-values for organic acids. For each metabolite, a Kruskal-Wallis test was performed to assess overall differences among groups 1 to 8 (0-12 months infant formulas), 9 HD cow's milk, 10 conventional cow's milk, 11 goat's milk, 12 human milk. When significant, pairwise comparisons were conducted using Dunn's post-hoc test, with p-values adjusted using the False Discovery Rate (FDR) method. The Cfr column indicates the pair of groups being compared (e.g., 1–2 denotes the comparison between group 1 and group 2). FDR-adjusted p-values < 0.05 are considered statistically significant and are highlighted in red.

**Cfr**	2-oxoglutarate	cis-aconitate	citrate	formate	fumarate	hippurate	lactate	succinate
1-2	0.481	0.800	0.005	0.973	0.668	0.105	0.056	0.529
1-3	0.037	0.551	0.131	0.119	0.280	0.015	0.360	0.117

2-3	0.183	0.716	0.348	0.145	0.112	0.290	0.423	0.022
1-4	0.183	0.551	0.059	0.973	0.650	0.705	0.056	0.887
2-4	0.677	0.716	0.297	0.973	0.978	0.189	0.833	0.537
3-4	0.259	0.900	0.920	0.104	0.075	0.018	0.502	0.058
1-5	0.839	0.631	1.000	0.799	0.664	0.664	0.952	0.680
2-5	0.327	0.816	0.005	0.706	0.412	0.297	0.056	0.793
3-5	0.022	0.852	0.131	0.060	0.475	0.038	0.340	0.046
4-5	0.132	0.893	0.059	0.706	0.346	0.915	0.055	0.725
1-6	0.042	0.430	0.059	0.209	0.846	0.828	0.055	0.466
2-6	0.183	0.551	0.667	0.247	0.586	0.290	0.755	0.185
3-6	0.950	0.791	0.667	0.908	0.417	0.040	0.304	0.537
4-6	0.259	0.701	0.667	0.190	0.559	0.927	0.611	0.364
5-6	0.028	0.685	0.059	0.125	0.844	0.892	0.052	0.278
1-7	0.566	0.716	0.565	0.973	0.417	0.828	0.773	0.657
2-7	0.183	0.893	0.059	0.998	0.668	0.235	0.147	0.293
3-7	0.017	0.802	0.431	0.156	0.051	0.029	0.599	0.312
4-7	0.058	0.816	0.316	0.973	0.641	0.908	0.157	0.537
5-7	0.720	0.893	0.565	0.706	0.233	0.856	0.755	0.417
6-7	0.020	0.617	0.235	0.271	0.411	0.972	0.103	0.728
1-8	0.035	0.802	0.221	0.973	0.824	0.509	0.157	0.521
2-8	0.180	0.621	0.235	0.973	0.513	0.509	0.755	0.192
3-8	0.950	0.462	0.825	0.146	0.412	0.080	0.653	0.431
4-8	0.241	0.425	0.729	0.998	0.475	0.752	0.865	0.404
5-8	0.020	0.490	0.221	0.775	0.846	0.854	0.147	0.293
6-8	0.950	0.339	0.556	0.247	0.978	0.752	0.586	0.887
7-8	0.017	0.585	0.570	0.973	0.323	0.738	0.304	0.820
1-9	0.020	0.088	0.101	0.107	0.152	0.045	0.295	0.158
2-9	0.079	0.153	0.542	0.096	0.067	0.662	0.004	0.039
3-9	0.651	0.265	0.825	0.003	0.742	0.741	0.056	1.000
4-9	0.132	0.159	0.837	0.073	0.048	0.083	0.004	0.093
5-9	0.017	0.167	0.101	0.176	0.323	0.134	0.304	0.065
6-9	0.729	0.462	0.825	0.011	0.296	0.134	0.004	0.543
7-9	0.017	0.159	0.348	0.104	0.038	0.103	0.188	0.355
8-9	0.677	0.076	0.667	0.111	0.286	0.238	0.018	0.466
1-10	0.183	0.232	0.011	0.147	0.048	0.015	0.323	0.039
2-10	0.566	0.355	0.920	0.125	0.019	0.297	0.004	0.007
3-10	0.512	0.585	0.395	0.004	0.475	0.961	0.057	0.677
4-10	0.818	0.462	0.379	0.105	0.007	0.019	0.004	0.013
5-10	0.132	0.462	0.011	0.257	0.120	0.039	0.340	0.012
6-10	0.481	0.814	0.729	0.016	0.117	0.045	0.004	0.314
7-10	0.067	0.462	0.097	0.142	0.007	0.033	0.218	0.146
8-10	0.481	0.159	0.304	0.151	0.108	0.083	0.018	0.215
9-10	0.249	0.551	0.584	0.856	0.726	0.753	0.865	0.686
1-11	0.554	0.346	0.389	0.214	0.286	0.045	0.943	0.022
2-11	0.950	0.462	0.102	0.176	0.117	0.741	0.083	0.005
3-11	0.183	0.714	0.587	0.009	0.978	0.609	0.436	0.543
4-11	0.644	0.610	0.482	0.153	0.081	0.083	0.085	0.007

5-11	0.432	0.585	0.389	0.408	0.475	0.149	0.911	0.007
6-11	0.183	0.908	0.355	0.029	0.417	0.149	0.059	0.245
7-11	0.241	0.551	0.804	0.194	0.054	0.112	0.833	0.097
8-11	0.180	0.232	0.729	0.214	0.417	0.288	0.205	0.158
9-11	0.079	0.462	0.482	0.704	0.726	0.892	0.294	0.570
10-11	0.549	0.836	0.145	0.908	0.468	0.652	0.321	0.861
1-12	0.950	0.159	0.001	0.038	0.323	0.664	0.640	0.215
2-12	0.481	0.255	0.618	0.029	0.545	0.039	0.018	0.537
3-12	0.042	0.462	0.144	0.000	0.038	0.004	0.173	0.007
4-12	0.191	0.355	0.101	0.016	0.475	0.297	0.018	0.206
5-12	0.905	0.355	0.001	0.096	0.142	0.297	0.653	0.411
6-12	0.045	0.699	0.395	0.003	0.318	0.509	0.018	0.058
7-12	0.644	0.346	0.018	0.038	0.846	0.509	0.457	0.098
8-12	0.037	0.147	0.101	0.043	0.265	0.235	0.057	0.058
9-12	0.020	0.690	0.297	0.891	0.024	0.019	0.596	0.010
10-12	0.183	0.822	0.584	0.600	0.007	0.004	0.653	0.002
11-12	0.549	0.714	0.050	0.406	0.038	0.019	0.611	0.001

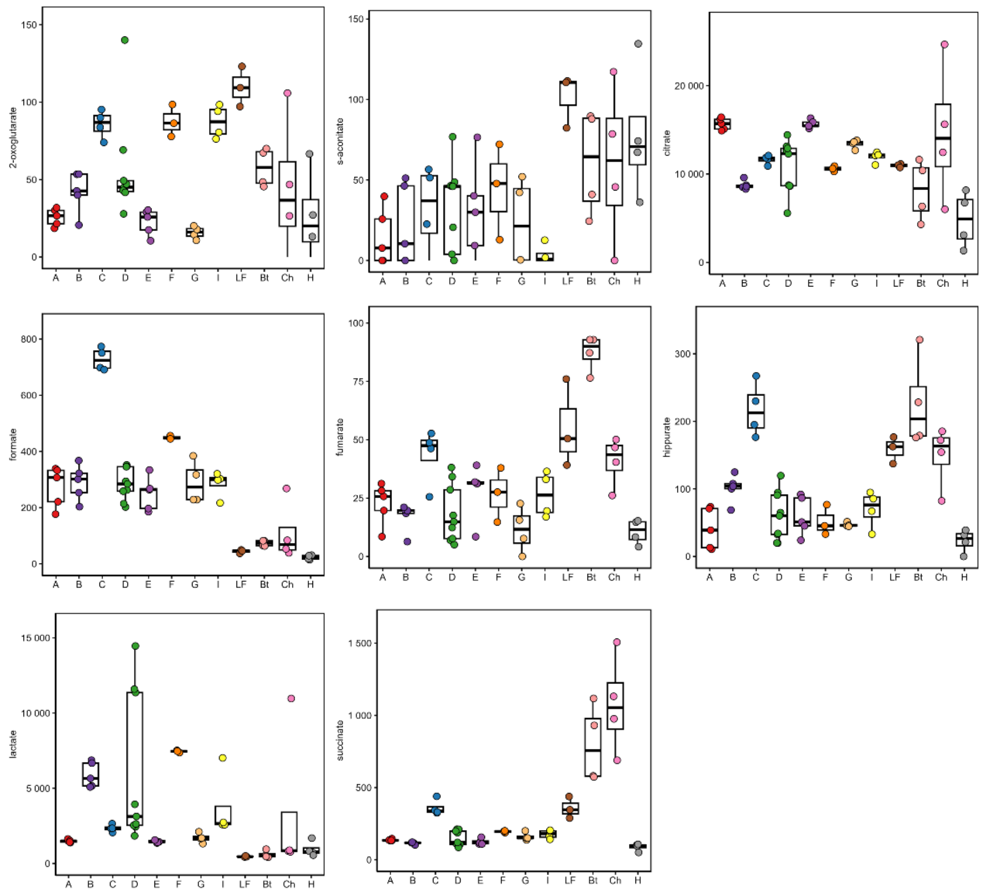

Figure S5. Boxplot of the levels of organic acids performed to assess overall differences among groups 1 to 8 or A to I (0-12 months infant formulas), 9 or LF (lactose free) HD cow's milk, 10 or Bt (Bos taurus) conventional cow's milk, 11 or Ch (Capra hircus) goat's milk, 12 of H (human milk).

Table S5. Table of p-values for amines and derivatives. For each metabolite, a Kruskal-Wallis test was performed to assess overall differences among groups 1 to 8 (0-12 months infant formulas), 9 HD cow's milk, 10 conventional cow's milk, 11 goat's milk, 12 human milk. When significant, pairwise comparisons were conducted using Dunn's post-hoc test, with p-values adjusted using the False Discovery Rate (FDR) method. The Cfr column indicates the pair of groups being compared (e.g., 1–2 denotes the comparison between group 1 and group 2). FDR-adjusted p-values < 0.05 are considered statistically significant and are highlighted in red.

**Cfr**	choline	creatine	creatinine+phosphocreatine	dimethylamine	methylamine	trimethylamine
1-2	0.191	0.110	0.132	0.239	0.831	0.211
1-3	0.290	0.091	0.002	0.004	0.148	0.016
2-3	0.015	0.867	0.132	0.092	0.079	0.257
1-4	0.505	0.123	0.003	0.346	0.715	0.769
2-4	0.022	0.788	0.310	0.785	0.869	0.267
3-4	0.537	0.666	0.493	0.024	0.033	0.016
1-5	0.984	0.867	0.962	0.854	0.773	0.721
2-5	0.193	0.135	0.145	0.142	0.578	0.100
3-5	0.289	0.123	0.002	0.003	0.323	0.004
4-5	0.504	0.166	0.004	0.198	0.363	0.418
1-6	0.984	0.110	0.132	0.115	0.473	0.073
2-6	0.294	0.845	0.854	0.639	0.314	0.471
3-6	0.341	0.950	0.284	0.350	0.770	0.776
4-6	0.521	0.666	0.519	0.386	0.166	0.093
5-6	0.984	0.135	0.137	0.081	0.725	0.019
1-7	0.390	0.683	0.981	0.024	0.531	0.379
2-7	0.752	0.277	0.137	0.274	0.725	0.776
3-7	0.052	0.242	0.002	0.639	0.033	0.176
4-7	0.093	0.349	0.005	0.120	0.787	0.523
5-7	0.390	0.791	0.948	0.011	0.303	0.209
6-7	0.478	0.253	0.133	0.715	0.140	0.328
1-8	0.119	0.499	0.493	0.388	0.787	0.456
2-8	0.752	0.396	0.489	0.854	0.961	0.725
3-8	0.009	0.349	0.029	0.071	0.079	0.151
4-8	0.015	0.502	0.068	0.970	0.940	0.626
5-8	0.119	0.609	0.545	0.274	0.560	0.257
6-8	0.184	0.349	0.429	0.471	0.309	0.267
7-8	0.531	0.815	0.493	0.198	0.773	0.935
1-9	0.505	0.012	0.064	0.008	0.079	0.257
2-9	0.064	0.292	0.588	0.121	0.043	0.964
3-9	0.752	0.396	0.442	0.970	0.773	0.334
4-9	0.886	0.154	0.804	0.052	0.023	0.337
5-9	0.505	0.018	0.072	0.004	0.166	0.148
6-9	0.517	0.455	0.804	0.388	0.527	0.533
7-9	0.142	0.042	0.068	0.693	0.023	0.776
8-9	0.038	0.078	0.276	0.099	0.043	0.729
1-10	0.390	0.009	0.002	0.004	0.057	0.359
2-10	0.026	0.292	0.132	0.114	0.033	0.776
3-10	0.852	0.396	0.981	0.970	0.773	0.185
4-10	0.752	0.135	0.489	0.032	0.020	0.494
5-10	0.390	0.012	0.002	0.003	0.144	0.197

6-10	0.434	0.465	0.278	0.388	0.518	0.334
7-10	0.085	0.035	0.002	0.693	0.022	0.964
8-10	0.015	0.067	0.029	0.085	0.033	0.913
9-10	0.903	0.937	0.433	0.970	0.967	0.776
1-11	0.120	0.002	0.002	0.004	0.033	0.492
2-11	0.004	0.132	0.132	0.114	0.023	0.689
3-11	0.726	0.208	1.000	0.970	0.647	0.138
4-11	0.325	0.043	0.493	0.032	0.015	0.689
5-11	0.119	0.003	0.002	0.003	0.079	0.261
6-11	0.174	0.266	0.284	0.388	0.358	0.257
7-11	0.015	0.012	0.002	0.693	0.015	0.890
8-11	0.002	0.024	0.029	0.085	0.023	0.957
9-11	0.505	0.782	0.442	0.970	0.901	0.699
10-11	0.537	0.683	0.981	1.000	0.869	0.866
1-12	0.064	0.591	0.361	0.236	0.814	0.471
2-12	0.002	0.035	0.679	0.970	0.967	0.058
3-12	0.513	0.031	0.064	0.120	0.085	0.002
4-12	0.174	0.035	0.135	0.722	0.911	0.257
5-12	0.064	0.482	0.405	0.142	0.578	0.769
6-12	0.104	0.036	0.545	0.693	0.314	0.013
7-12	0.008	0.349	0.366	0.350	0.773	0.138
8-12	0.002	0.245	0.804	0.808	0.967	0.159
9-12	0.390	0.003	0.405	0.154	0.046	0.093
10-12	0.405	0.002	0.064	0.142	0.035	0.138
11-12	0.791	0.001	0.064	0.142	0.024	0.176

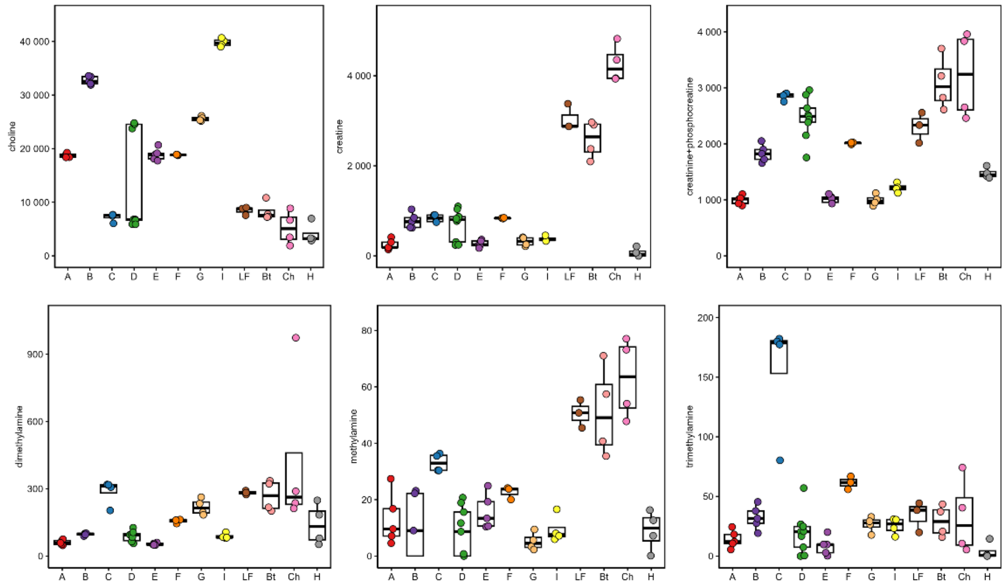

Figure S6. Boxplot of the levels of amines and derivatives performed to assess overall differences among groups 1 to 8 or A to I (0-12 months infant formulas), 9 or LF (lactose free) HD cow's milk, 10 or Bt (Bos taurus) conventional cow's milk, 11 or Ch (Capra hircus) goat's milk, 12 of H (human milk).

Table S6. Table of p-values for vitamins. For each metabolite, a Kruskal-Wallis test was performed to assess overall differences among groups 1 to 8 (0-12 months infant formulas), 9 HD cow's milk, 10 conventional cow's milk, 11 goat's milk, 12 human milk . When significant, pairwise comparisons were conducted using Dunn's post-hoc test, with p-values adjusted using the False Discovery Rate (FDR) method. The Cfr column indicates the pair of groups being compared (e.g., 1–2 denotes the comparison between group 1 and group 2). FDR-adjusted p-values < 0.05 are considered statistically significant and are highlighted in red.

**Cfr**	ascorbate	niacinamide
1-2	0.916	0.044
1-3	0.295	0.989
2-3	0.343	0.059
1-4	0.113	0.071
2-4	0.107	0.613
3-4	0.008	0.095
1-5	0.968	0.648
2-5	0.912	0.098
3-5	0.288	0.654
4-5	0.116	0.222
1-6	0.474	0.427
2-6	0.400	0.356
3-6	0.107	0.427
4-6	0.730	0.567
5-6	0.477	0.732
1-7	0.337	0.427
2-7	0.288	0.253
3-7	0.067	0.444
4-7	0.785	0.427
5-7	0.343	0.793
6-7	0.916	0.934
1-8	0.769	0.323
2-8	0.710	0.386
3-8	0.162	0.348
4-8	0.323	0.648
5-8	0.785	0.577
6-8	0.768	0.903
7-8	0.658	0.823
1-9	0.198	0.348
2-9	0.239	0.003
3-9	0.837	0.372
4-9	0.008	0.005
5-9	0.191	0.151
6-9	0.075	0.098
7-9	0.042	0.098
8-9	0.113	0.059
1-10	0.113	0.381
2-10	0.135	0.003
3-10	0.713	0.401
4-10	0.001	0.004
5-10	0.113	0.162

6-10	0.042	0.098
7-10	0.016	0.098
8-10	0.075	0.059
9-10	0.912	0.903
1-11	0.107	0.375
2-11	0.116	0.003
3-11	0.663	0.396
4-11	0.001	0.004
5-11	0.107	0.159
6-11	0.037	0.098
7-11	0.014	0.098
8-11	0.067	0.059
9-11	0.896	0.903
10-11	0.943	0.989
1-12	0.912	0.222
2-12	0.843	0.002
3-12	0.242	0.253
4-12	0.209	0.002
5-12	0.912	0.094
6-12	0.635	0.059
7-12	0.477	0.059
8-12	0.896	0.032
9-12	0.162	0.903
10-12	0.107	0.794
11-12	0.107	0.799

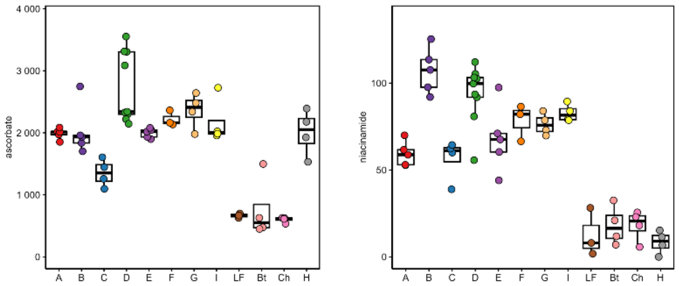

Figure S7. Boxplot of the levels of vitamins performed to assess overall differences among groups 1 to 8 or A to I (0-12 months infant formulas), 9 or LF (lactose free) HD cow's milk, 10 or Bt (Bos taurus) conventional cow's milk, 11 or Ch (Capra hircus) goat's milk, 12 of H (human milk).

Table S7. Table of p-values for carbohydrates and sugars. For each metabolite, a Kruskal-Wallis test was performed to assess overall differences among groups 1 to 8 (0-12 months infant formulas), 9 HD cow's milk, 10 conventional cow's milk, 11 goat's milk, 12 human milk . When significant, pairwise comparisons were conducted using Dunn's post-hoc test, with p-values adjusted using the False Discovery Rate (FDR) method. The Cfr column indicates the pair of groups being compared (e.g., 1–2

denotes the comparison between group 1 and group 2). FDR-adjusted p-values < 0.05 are considered statistically significant and are highlighted in red.

**Cfr**	2'-fucosyllactose	3'-fucosyllactose	arabinose	fucose	Fucosyl-α-1,3-N-acetylglucosamine	Fucosyl-α-1,4-N-acetylglucosamine	galactose	glucose	lactose	lactulose	maltodextrin	mannose	N-acetyl carbohydrates	raffinose	UDP-galactose	UDP-glucose
1-2	1	$6.8\cdot10^{-3}$	0.672	0.190	0.059	0.249	0.220	0.006	0.290	0.628	0.046	0.091	0.105	0.011	1	0.838
1-3	1	$6.8\cdot10^{-3}$	0.134	0.012	0.816	0.274	0.431	0.006	0.106	0.090	0.210	0.024	0.329	0.745	0.880	0.838
2-3	1	1	0.365	0.249	0.036	0.032	0.047	1	0.007	0.029	0.001	0.586	0.624	0.003	0.880	0.984
1-4	0.137	0.361	0.400	0.111	0.272	0.883	0.130	0.029	0.418	0.611	1	0.019	0.011	0.574	0.935	0.370
2-4	0.137	0.035	0.134	0.948	0.320	0.274	0.919	0.434	0.034	0.304	0.025	0.652	0.496	0.031	0.977	0.838
3-4	0.163	0.049	0.009	0.224	0.185	0.185	0.020	0.449	0.348	0.139	0.140	0.824	0.231	0.213	0.977	0.838
1-5	1	1	0.593	0.952	0.910	0.920	0.900	1	0.783	0.984	1	1	0.859	0.861	1	0.838
2-5	1	$6.8\cdot10^{-3}$	0.999	0.179	0.074	0.315	0.171	0.006	0.184	0.624	0.038	0.091	0.165	0.006	1	0.984
3-5	1	$6.8\cdot10^{-3}$	0.400	0.012	0.701	0.229	0.456	0.006	0.193	0.090	0.234	0.024	0.411	0.809	0.880	0.984
4-5	0.137	0.211	0.134	0.098	0.345	0.950	0.104	0.021	0.590	0.611	0.963	0.019	0.019	0.400	0.977	0.838
1-6	0.047	0.012	0.957	0.363	0.439	0.883	0.210	0.013	0.830	0.324	0.329	0.055	0.003	0.227	0.880	0.370
2-6	0.047	1	0.593	0.803	0.405	0.454	0.845	1	0.264	0.608	0.412	0.691	0.159	0.499	0.890	0.838
3-6	0.053	1	0.134	0.224	0.345	0.249	0.047	1	0.230	0.014	0.046	0.907	0.058	0.092	1	0.838
4-6	0.501	0.07	0.593	0.735	0.917	0.950	0.880	0.463	0.614	0.122	0.316	0.966	0.329	0.574	0.977	0.898
5-6	0.047	$6.8\cdot10^{-3}$	0.550	0.363	0.500	0.950	0.167	0.010	0.949	0.322	0.295	0.055	0.007	0.153	0.880	0.838
1-7	0.014	$6.8\cdot10^{-3}$	0.316	0.073	0.467	0.260	0.700	0.006	0.745	0.308	0.523	0.170	0.034	0.861	1	0.838
2-7	0.014	1	0.134	0.591	0.010	0.029	0.104	1	0.538	0.126	0.190	0.905	0.573	0.029	1	0.984
3-7	0.017	1	0.009	0.577	0.701	0.950	0.701	1	0.060	0.590	0.069	0.480	0.329	0.574	0.880	0.984
4-7	0.207	0.049	0.746	0.591	0.068	0.162	0.058	0.449	0.239	0.559	0.523	0.586	0.981	0.809	0.891	0.894
5-7	0.014	$6.8\cdot10^{-3}$	0.134	0.071	0.417	0.228	0.742	0.006	0.581	0.308	0.494	0.170	0.053	0.800	1	0.984
6-7	1	1	0.466	0.516	0.181	0.235	0.104	1	0.633	0.061	0.769	0.606	0.374	0.400	0.880	0.838
1-8	1	1	0.316	0.612	0.449	0.792	0.731	0.692	0.106	0.590	0.096	0.691	0.774	0.039	0.880	0.838
2-8	1	$6.8\cdot10^{-3}$	0.134	0.462	0.320	0.465	0.436	0.037	0.581	0.308	0.782	0.294	0.231	0.809	0.880	0.984
3-8	1	$6.8\cdot10^{-3}$	0.009	0.071	0.345	0.206	0.253	0.043	0.002	0.308	0.005	0.083	0.529	0.011	1	0.984
4-8	0.162	0.306	0.746	0.363	0.859	0.920	0.353	0.207	0.007	0.862	0.078	0.079	0.045	0.140	0.977	0.838
5-8	1	1	0.134	0.591	0.520	0.920	0.678	0.624	0.056	0.593	0.086	0.691	0.910	0.021	0.880	0.984
6-8	0.053	0.012	0.466	0.637	0.917	0.950	0.403	0.066	0.106	0.131	0.653	0.163	0.014	0.749	1	0.838
7-8	0.017	$6.8\cdot10^{-3}$	1	0.224	0.176	0.186	0.456	0.043	0.230	0.659	0.343	0.426	0.092	0.099	0.880	0.981
1-9	1	0.012	0.369	0.363	0.272	0.105	0.057	0.692	0.047	0.100	0.243	0.019	0.063	0.767	0.880	0.634
2-9	1	1	0.140	0.033	0.005	0.008	0.436	0.006	0.307	0.229	0.003	0.386	0.712	0.007	0.890	0.838
3-9	1	1	0.019	0.003	0.405	0.525	0.010	0.006	0.001	0.002	1	0.784	0.405	1	1	0.838
4-9	0.22	0.07	0.776	0.014	0.026	0.043	0.436	0.013	0.004	0.023	0.190	0.587	0.910	0.281	0.977	0.984
5-9	1	$6.8\cdot10^{-3}$	0.134	0.363	0.220	0.075	0.047	0.763	0.026	0.100	0.279	0.019	0.103	0.824	0.880	0.838
6-9	0.073	1	0.497	0.097	0.077	0.098	0.626	0.006	0.050	0.608	0.068	0.691	0.369	0.119	1	0.983
7-9	0.032	1	1	0.012	0.656	0.555	0.027	0.006	0.106	0.013	0.090	0.317	0.917	0.622	0.880	0.898
8-9	1	0.012	1	0.190	0.074	0.062	0.130	0.434	0.649	0.033	0.010	0.055	0.159	0.021	1	0.838
1-10	1	$6.8\cdot10^{-3}$	0.718	0.626	0.272	0.315	0.628	0.082	0.230	0.139	0.210	0.161	0.015	0.745	0.880	0.838
2-10	1	1	1	0.083	0.003	0.041	0.096	0.460	0.830	0.326	0.001	0.935	0.380	0.003	0.880	0.984
3-10	1	1	0.369	0.005	0.417	0.950	0.742	0.460	0.007	0.002	1	0.520	0.199	1	0.520	0.984

4-10	0.162	0.049	0.167	0.047	0.021	0.216	0.047	1	0.027	0.033	0.140	0.586	0.774	0.213	0.541	0.838
5-10	1	$6.8\cdot10^{-3}$	0.995	0.648	0.220	0.255	0.701	0.066	0.132	0.139	0.234	0.161	0.023	0.809	0.880	0.984
6-10	0.053	1	0.632	0.209	0.077	0.263	0.096	0.494	0.219	0.744	0.046	0.644	0.551	0.092	0.541	0.838
7-10	0.017	1	0.134	0.030	0.701	0.943	0.919	0.460	0.421	0.016	0.069	0.966	0.786	0.574	0.880	0.984
8-10	1	$6.8\cdot10^{-3}$	0.134	0.363	0.074	0.228	0.436	0.376	0.725	0.056	0.005	0.391	0.046	0.011	0.520	0.984
9-10	1	1	0.167	0.612	0.917	0.465	0.021	0.039	0.442	0.744	1	0.345	0.734	1	0.541	0.838
1-11	1	$6.8\cdot10^{-3}$	0.316	0.577	0.135	0.436	0.100	0.006	0.060	0.953	0.210	0.586	0.369	0.745	0.006	0.015
2-11	1	1	0.134	0.527	0.001	0.062	0.005	1	0.439	0.611	0.001	0.026	0.573	0.003	0.005	0.077
3-11	1	1	0.009	0.083	0.272	0.883	0.456	1	0.001	0.118	1	0.010	0.943	1	0.002	0.077
4-11	0.162	0.049	0.746	0.429	0.006	0.260	0.001	0.449	0.004	0.659	0.140	0.008	0.200	0.213	0.002	0.136
5-11	1	$6.8\cdot10^{-3}$	0.134	0.555	0.097	0.315	0.122	0.006	0.034	0.954	0.234	0.586	0.465	0.809	0.005	0.077
6-11	0.053	1	0.466	0.695	0.036	0.329	0.007	1	0.065	0.322	0.046	0.021	0.053	0.092	0.003	0.634
7-11	0.017	1	1	0.249	0.450	0.870	0.253	1	0.164	0.345	0.069	0.055	0.296	0.574	0.013	0.116
8-11	1	$6.8\cdot10^{-3}$	1	0.939	0.031	0.260	0.056	0.043	0.830	0.611	0.005	0.353	0.573	0.011	0.002	0.077
9-11	1	1	1	0.169	0.859	0.362	0.001	0.006	0.783	0.100	1	0.008	0.374	1	0.003	0.370
10-11	1	1	0.134	0.343	0.743	0.920	0.296	0.460	0.590	0.139	1	0.052	0.170	1	0.129	0.079
1-12	$6.6\cdot10^{-3}$	0.60	0.746	0.249	0.087	0.098	0.220	0.006	0.421	0.061	0.523	0.966	0.001	0.928	0.880	0.575
2-12	$6.6\cdot10^{-3}$	0.059	0.466	0.012	0.910	0.555	0.017	1	0.058	0.139	0.190	0.136	0.058	0.011	0.880	0.838
3-12	$6.6\cdot10^{-3}$	0.069	0.134	0.001	0.068	0.008	0.731	1	0.518	0.001	0.069	0.035	0.022	0.809	1	0.838
4-12	0.071	1	0.718	0.005	0.417	0.105	0.005	0.449	0.871	0.010	0.523	0.026	0.165	0.499	0.977	0.984
5-12	$6.6\cdot10^{-3}$	0.42	0.400	0.267	0.122	0.153	0.272	0.006	0.590	0.061	0.494	0.966	0.001	1	0.880	0.838
6-12	0.7961	0.09	0.935	0.063	0.461	0.228	0.021	1	0.590	0.550	0.769	0.079	0.848	0.207	1	0.978
7-12	1	0.069	0.550	0.005	0.023	0.008	0.456	1	0.264	0.005	1	0.218	0.231	0.809	0.880	0.898
8-12	$6.6\cdot10^{-3}$	0.51	0.550	0.112	0.405	0.228	0.130	0.043	0.017	0.016	0.343	0.751	0.003	0.033	1	0.838
9-12	0.014	0.09	0.583	0.926	0.008	0.003	0.005	0.006	0.007	0.953	0.090	0.024	0.231	0.809	1	0.984
10-12	$6.6\cdot10^{-3}$	0.69	0.486	0.540	0.006	0.009	0.519	0.460	0.047	0.659	0.069	0.196	0.374	0.809	0.520	0.838
11-12	$6.6\cdot10^{-3}$	0.69	0.550	0.097	0.003	0.017	0.701	1	0.008	0.063	0.069	0.586	0.019	0.809	0.002	0.334

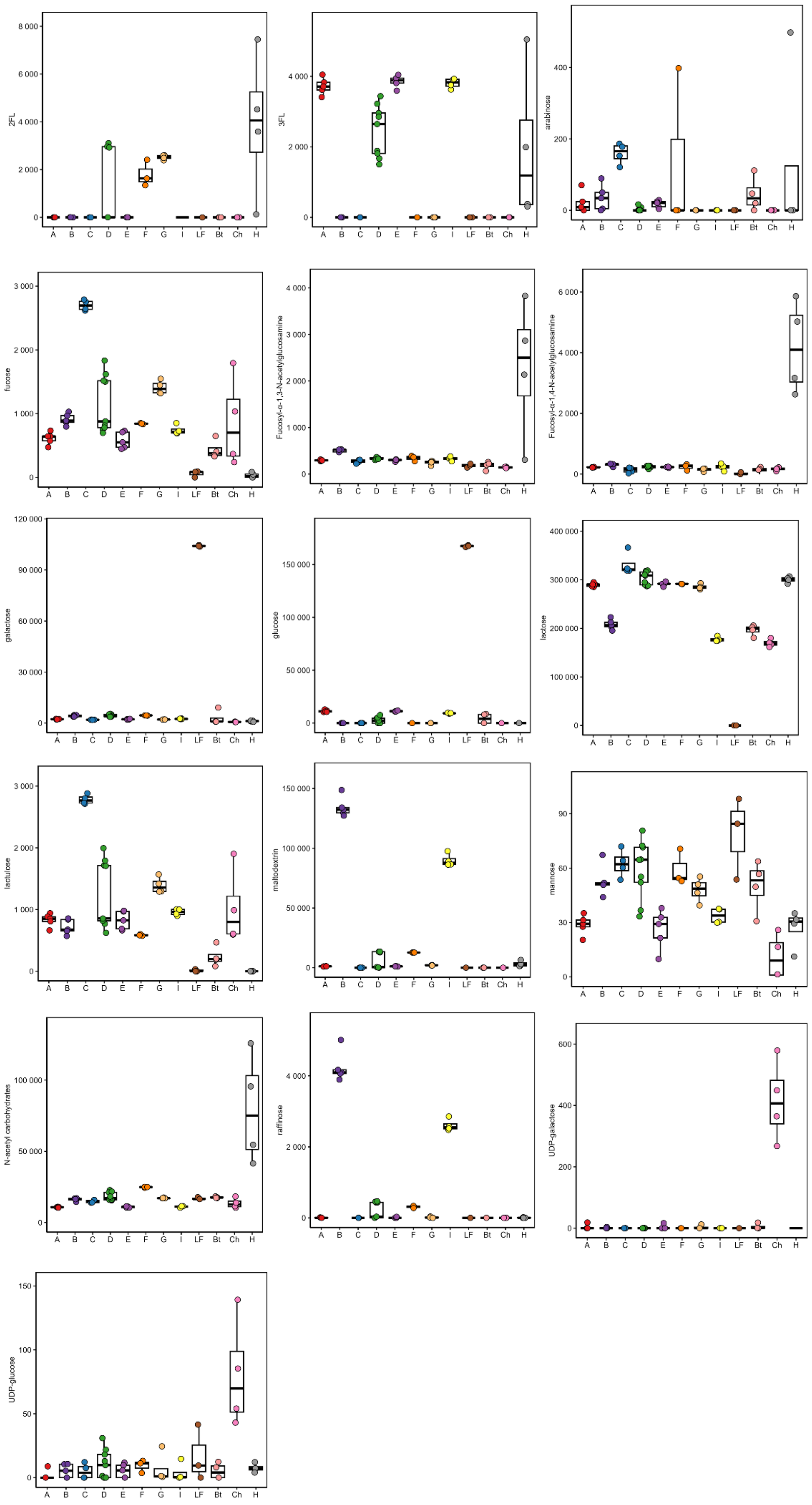
2FL
3FL
arabinose
fucose
Fucosyl-α-1,3-N-acetylglucosamine
Fucosyl-α-1,4-N-acetylglucosamine
galactose
glucose
lactose
lactulose
maltodextrin
mannose
N-acetyl carbohydrates
raffinose
UDP-galactose
UDP-glucose
A B C D E F G I LF Bt Ch H

Figure S8. Boxplot of the levels of carbohydrates and sugars performed to assess overall differences among groups 1 to 8 or A to I (0-12 months infant formulas), 9 or LF (lactose free) HD cow's milk, 10 or Bt (Bos taurus) conventional cow's milk, 11 or Ch (Capra hircus) goat's milk, 12 of H (human milk).

Table S8. Table of p-values for energetic compounds, short chain fatty acids (SCFA), nucleotides and derivatives and other compounds. For each metabolite, a Kruskal-Wallis test was performed to assess overall differences among groups 1 to 8 (0-12 months infant formulas), 9 HD cow's milk, 10 conventional cow's milk, 11 goat's milk, 12 human milk . When significant, pairwise comparisons were conducted using Dunn's post-hoc test, with p-values adjusted using the False Discovery Rate (FDR) method. The Cfr column indicates the pair of groups being compared (e.g., 1–2 denotes the comparison between group 1 and group 2). FDR-adjusted p-values < 0.05 are considered statistically significant and are highlighted in red.

	Energetic compounds			SCFA		Nucleotides and derivatives			Other compounds		
cfr	acetyl-carnitine	carnitine	glycerophosphocholine	acetate	butyrate	inosine	uridine	orotate	acetone	dimethylsulfone	ethanol
1-2	0.008	0.062	0.010	0.047	0.662	0.064	0.137	0.883	0.675	0.360	1
1-3	0.608	0.206	0.121	0.003	0.062	0.046	0.416	0.049	0.105	0.014	1
2-3	0.077	0.652	0.531	0.315	0.187	0.914	0.598	0.038	0.037	0.125	1
1-4	0.008	0.027	0.000	0.004	0.608	0.842	0.659	0.664	0.398	0.058	1
2-4	0.755	0.905	0.549	0.616	0.955	0.014	0.023	0.539	0.155	0.497	1
3-4	0.086	0.560	0.152	0.558	0.147	0.014	0.169	0.083	0.333	0.360	1
1-5	0.983	0.809	0.943	0.902	0.955	0.922	0.996	0.886	0.949	0.873	1
2-5	0.008	0.033	0.013	0.035	0.676	0.046	0.137	0.780	0.709	0.258	1
3-5	0.599	0.155	0.145	0.003	0.067	0.036	0.416	0.064	0.093	0.008	1
4-5	0.008	0.011	0.000	0.003	0.641	0.922	0.659	0.744	0.337	0.031	1
1-6	0.133	0.485	0.001	0.023	0.433	0.662	0.659	0.173	0.113	0.058	1
2-6	0.540	0.421	0.294	0.616	0.651	0.382	0.078	0.136	0.043	0.360	1
3-6	0.421	0.719	0.092	0.701	0.489	0.293	0.209	0.724	0.949	0.726	1
4-6	0.620	0.338	0.566	0.867	0.649	0.442	0.897	0.265	0.333	0.669	1
5-6	0.128	0.357	0.001	0.017	0.462	0.598	0.659	0.205	0.103	0.036	1
1-7	0.617	0.730	0.251	0.538	0.310	0.082	0.996	0.225	0.182	0.012	1
2-7	0.072	0.176	0.282	0.246	0.541	0.998	0.165	0.173	0.071	0.113	1
3-7	0.957	0.421	0.701	0.035	0.550	0.917	0.433	0.536	0.815	0.956	1
4-7	0.077	0.111	0.058	0.072	0.505	0.025	0.673	0.368	0.544	0.342	1
5-7	0.611	0.584	0.285	0.466	0.315	0.057	0.996	0.270	0.158	0.007	1
6-7	0.387	0.699	0.043	0.121	0.892	0.398	0.659	0.791	0.769	0.698	1
1-8	0.302	0.905	0.566	0.292	0.550	0.293	0.416	0.492	0.092	0.889	1
2-8	0.241	0.062	0.083	0.472	0.837	0.693	0.611	0.405	0.029	0.486	1
3-8	0.617	0.196	0.371	0.092	0.310	0.574	0.996	0.265	0.949	0.028	1
4-8	0.314	0.029	0.008	0.201	0.837	0.107	0.169	0.722	0.298	0.114	1
5-8	0.291	0.905	0.605	0.246	0.582	0.215	0.416	0.551	0.075	0.726	1
6-8	0.667	0.437	0.008	0.246	0.823	0.694	0.208	0.515	0.994	0.104	1
7-8	0.608	0.689	0.566	0.701	0.663	0.693	0.416	0.664	0.769	0.025	1
1-9	0.008	0.196	0.069	0.671	0.033	0.116	0.203	0.035	0.337	0.004	1
2-9	0.795	0.772	0.761	0.238	0.071	0.998	0.991	0.026	0.161	0.036	1
3-9	0.072	0.905	0.701	0.035	0.672	0.917	0.659	0.744	0.675	0.565	1
4-9	0.608	0.699	0.397	0.070	0.060	0.045	0.085	0.049	0.769	0.107	1
5-9	0.008	0.147	0.078	0.616	0.033	0.086	0.203	0.043	0.315	0.002	1
6-9	0.426	0.652	0.246	0.111	0.310	0.440	0.137	0.539	0.647	0.380	1
7-9	0.072	0.379	0.540	0.867	0.333	0.998	0.209	0.370	0.815	0.583	1
8-9	0.186	0.187	0.240	0.616	0.154	0.705	0.659	0.173	0.627	0.008	1
1-10	0.072	0.012	0.007	0.041	0.033	0.057	0.802	0.018	0.287	0.002	3.63·10-6

2-10	0.611	0.542	0.682	0.867	0.068	0.922	0.224	0.014	0.559	0.028	3.63·10-6
3-10	0.279	0.272	0.292	0.446	0.743	0.963	0.633	0.695	0.009	0.565	7.3·10-6
4-10	0.770	0.542	0.851	0.737	0.051	0.014	0.490	0.026	0.037	0.084	4.81·10-7
5-10	0.072	0.007	0.008	0.035	0.033	0.045	0.802	0.026	0.315	0.002	3.63·10-6
6-10	0.857	0.176	0.552	0.701	0.315	0.329	0.490	0.482	0.011	0.374	3.52·10-5
7-10	0.241	0.049	0.152	0.238	0.338	0.922	0.802	0.281	0.016	0.583	7.3·10-6
8-10	0.548	0.012	0.043	0.410	0.154	0.618	0.611	0.127	0.009	0.005	7.3·10-6
9-10	0.527	0.410	0.552	0.201	0.920	0.922	0.334	0.923	0.054	0.956	3.52·10-5
1-11	0.540	0.002	0.566	0.297	0.837	0.364	0.169	0.395	0.282	0.002	0.917
2-11	0.086	0.216	0.084	0.466	0.550	0.006	0.002	0.482	0.552	0.023	0.917
3-11	0.935	0.111	0.376	0.091	0.051	0.006	0.023	0.008	0.009	0.497	0.917
4-11	0.106	0.199	0.008	0.199	0.489	0.448	0.241	0.167	0.037	0.058	0.917
5-11	0.535	0.002	0.598	0.246	0.833	0.440	0.169	0.329	0.310	0.002	0.917
6-11	0.477	0.061	0.008	0.245	0.338	0.153	0.474	0.035	0.011	0.342	0.946
7-11	0.875	0.011	0.566	0.701	0.250	0.010	0.185	0.043	0.016	0.536	0.917
8-11	0.677	0.002	0.982	0.982	0.464	0.035	0.023	0.135	0.009	0.004	0.917
9-11	0.080	0.185	0.242	0.616	0.033	0.014	0.013	0.006	0.053	0.956	0.946
10-11	0.314	0.652	0.043	0.405	0.033	0.006	0.137	0.002	0.994	0.935	0.001
1-12	0.095	0.002	0.240	0.581	0.281	0.014	0.322	0.202	0.105	0.486	1
2-12	0.524	0.187	0.302	0.012	0.489	0.608	0.670	0.260	0.282	0.889	1
3-12	0.378	0.087	0.760	0.001	0.597	0.754	0.897	0.002	0.004	0.108	1
4-12	0.611	0.176	0.069	0.001	0.462	0.006	0.137	0.052	0.009	0.408	1
5-12	0.091	0.002	0.251	0.616	0.303	0.013	0.323	0.167	0.120	0.368	1
6-12	0.989	0.049	0.052	0.005	0.837	0.121	0.169	0.015	0.006	0.323	1
7-12	0.331	0.009	0.943	0.245	0.934	0.633	0.334	0.018	0.008	0.102	1
8-12	0.629	0.002	0.552	0.111	0.630	0.310	0.909	0.049	0.004	0.583	1
9-12	0.414	0.163	0.552	0.358	0.368	0.657	0.777	0.002	0.016	0.032	1
10-12	0.857	0.570	0.179	0.012	0.394	0.694	0.490	0.001	0.683	0.025	7.3·10-6
11-12	0.424	0.905	0.552	0.112	0.202	0.003	0.019	0.719	0.689	0.020	0.917

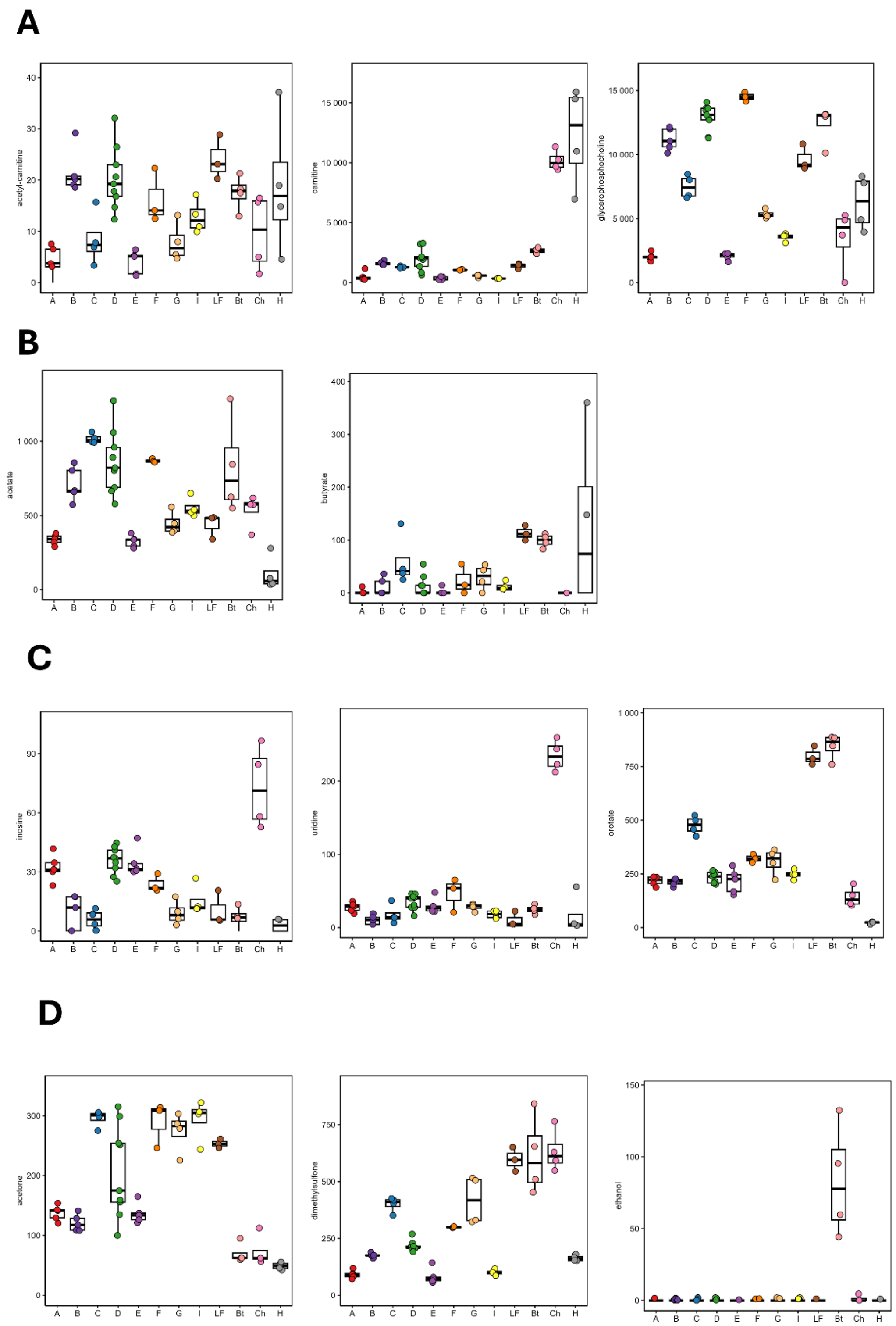

Figure S9. Boxplot of the levels of energetic compounds (acetyl-carnitine, carnitine and glycerophosphocholine). Panel A; SCFA (acetate and butyrate), panel B; nucleotides and derivatives (inosine, uridine and orotate), panel C; other compounds (acetone, dimethylsulfone and ethanol), panel D. Boxplot are performed to assess overall differences among groups 1 to 8 or A to I (0-12 months infant formulas), 9 or LF (lactose free) HD cow's milk, 10 or Bt (Bos taurus) conventional cow's milk, 11 or Ch (Capra hircus) goat's milk, 12 of H (human milk).

Table S9. Metabolites' mean ± standard deviations of 0-12 months and 1-3 years formula milk (brands from 1 to 8), lactose free cow milk (9), cow milk (10), goat milk (11) and human milk (12).

	1		2		3		4		5		6		7		8		9-10	11	12
	0-12m	1-3y	0-12m	1-3y	0-12m	1-3y	0-12m	1-3y	0-12m	1-3y	0-12m	1-3y	0-12m	1-3y	0-12m	1-3y			
**Amino acids**																			
alanine	990.9±47.8	543.1±95.9	1162.9±92.8	1193.7±78.4	915.6±73.7	761.7±53.9	1243.2±204.4	470.9±136.9	1008.4±43.5	500.4±53.1	1339.6±27.1	827.1±32.9	811.5±25.2	0±0	1134.1±47.7	552.9±4.2	803.8±82.7	1219±263.1	1142.5±194.4
glutamine	72.2±20.9	24.4±12.8	66.5±14.7	69.3±22.6	89.8±24.3	81.9±19.1	83.5±16.9	49.3±18.6	59.6±20.3	57.2±24	116.5±11.5	23.6±13.2	40.9±26.4	13.5±16.7	108.7±2.9	81.8±21.3	32.8±26	105.4±56.8	452.4±254.2
glutamate	73.3±10.6	193.5±36.3	113.7±9.4	209.6±30.1	357.7±10.3	367.3±14.8	161.2±28.5	236.3±55.7	90.3±12.1	180.7±35.6	156.6±4.5	259.6±6.8	70.8±4.6	84.3±10.4	87.7±12.7	272.3±7.6	560.4±32.3	261.8±19.5	2712.6±763.5
histidine	48.3±36.1	35.2±42.1	38.1±12.1	36±20.7	46.4±18.6	19.5±28.6	164.5±117	39.7±26.8	68.7±43.3	33.2±37.3	53.5±47.7	35.5±29.1	336.3±29.8	466.8±47.1	46.6±36.9	55.4±19.8	60.9±25.6	270.4±118.9	26±32.6
isoleucine	7.9±17.6	9.7±21.6	27.1±28	0±0	0±0	10.9±21.7	7.6±22.9	3.3±9.4	4.8±10.8	3.3±7.4	0±0	0±0	8.2±9.7	2846.8±11.7	13.7±19.9	0±0	1.5±4.1	33±66	0±0
leucine	163.2±29.1	18.5±20.2	156.2±16.9	143.4±29.3	151.9±18.8	114.1±15.6	207.7±37.7	0±0	161.4±35.4	0±0	233.8±11.6	99±1.4	143.4±21.1	1116.6±812.5	141.2±22.5	34.4±39.4	26.3±45	74.9±52	0±0
phenylalanine	23±19.2	0±0	4.6±8.3	20.8±17.5	1.9±3.8	10.7±12.4	1173.5±1101.3	10.8±29.6	27.4±34.6	23.3±32.1	24.6±25.7	9.5±3.9	5893.7±78.7	7609.4±209.3	8±16.1	4.9±8.5	1.6±4.3	16.7±19.3	73.6±58
tryptophan	10.4±10.1	17.6±20.2	15.1±19.8	12.6±16.8	12.1±7	28.2±14.4	167.1±134.9	19.8±16.3	21.3±15.1	4.1±9.2	19±26.2	27.5±36.6	18±14.8	429.7±44.7	12.4±17.8	18.4±15.9	20.4±15.2	14.7±17.9	12.8±8.8
tyrosine	25.7±18.5	20.6±25.8	25.1±16.9	14.6±14.4	9.2±10	6.9±10	1690.3±1023.3	29.6±22.1	17.7±26.4	18.6±14	13.2±13.3	9.7±9.2	50.8±15.7	25.8±23.2	20.2±35.9	15.2±4.9	19±19.8	50.1±19.6	31.2±46.7
valine	137.3±36.8	57.8±32	95.2±39.6	116.7±68.6	108.3±25.3	123.5±8.7	188.3±70.9	84.4±29.1	124.6±30.3	67.1±28.4	163.4±5.2	94.4±37.7	62.2±43.8	2167.5±48.2	55.6±28.7	81.1±11.6	204.1±88.4	654.1±161.8	419±165.7
**Organic acids**																			
2-oxoglutarate	25.7±5.6	34.5±13	42.1±13.5	54.4±14.1	85.7±9.1	66±19.7	56.2±33.2	58.4±27.9	22.6±8.4	33.4±14.9	87.6±10.3	32.9±3.2	15.8±4.1	4.9±8.5	87.3±10.6	56.9±13.4	80.1±30.2	44.8±45	26.7±28.8
cis-aconitate	14.7±17.6	27.8±31.7	21.6±25.2	25.5±36.9	32.6±26.4	47.7±23.9	32±27.1	56.6±25.9	31.2±29.9	27.1±39.3	44.3±29.8	71.4±22.8	23.7±27.4	32±37.9	3.6±6.1	60.6±6.3	78.2±33.4	60.4±49.7	78±41.2
citrate	15663.6±662.9	6889.4±2177.6	8748.2±494.1	15890.8±714.4	11634.7±510.2	11300.6±234	10780.2±2944.2	15463.7±4759.1	15644.6±458.2	7911.4±800	10599.8±304	18312.3±1355.3	13404.8±495.4	11356.9±2649	11935.9±627.9	12071.7±170.8	9365.5±2852.1	14687.5±7762.4	4838.5±3169.5
formate	275.2±72.4	482.1±33	289.5±63.2	284.6±34.3	728.2±40.2	369.2±120.4	284.4±56	189.1±23.6	249.4±60.3	527.1±37.3	449.3±6	105.6±11.7	289.7±75.7	335.7±89.3	284.3±46	302.2±60.8	60.8±17.4	111±106.4	23±8
fumarate	22.7±9	34.6±13.7	17.1±6.1	36±9.5	43.3±12.1	43.4±5.3	18.4±12.3	45.9±12.8	28.4±11.6	39.2±6.5	26.8±11.6	40.5±9.1	11.5±9.8	16.6±13.5	26.5±9.7	43.7±15.6	73.6±21.1	40.8±10.6	10.6±5.3
hippurate	41.4±	105±46.4	101.1±20.4	138.1±	217.2±40.1	251.1±	59.6±36	103.8±	59.7±28.8	102.1±	51.7±22.4	101.6±	46.9±3.1	68.8±30	70±27.3	107.2±	197.3±60.9	148.6±	23±17

	30.3			32.1		48.4		38.2		22.8		26.4				21.5		45.9	
lactate	1489.6±95.9	3337.1±268.6	5887.4±837.9	5979.9±1027.9	2341.7±241.1	2821.9±716.5	5990.3±4966.6	1382.7±1556.1	1460.1±82	3681.5±213.1	7451.5±74.2	1620.9±86.8	1694.8±323.6	4471.8±1000.3	3729.3±2192.8	3813±131	540.1±186.1	3364.2±5067.1	947.7±506.3
succinate	136.5±5.8	154.1±17.3	115.2±7.2	151.9±15	359.4±53.8	252.7±7.6	147.7±52.8	153.9±18.3	124.9±19.5	171.1±24.7	195±6.5	198.6±6.9	160±27.4	206.4±27.3	176.9±30	176.8±14.6	610.8±306.6	1075.8±340.7	89.1±25.6
**Amines and derivatives**																			
choline	18747.7±375	4123.4±194.3	32719.4±760.3	4877.8±361.4	7212.7±773	7205.6±1439.2	14418.1±9509.5	3796.1±200.3	18917.1±1134.8	4041±156.4	18833.3±80.8	4494.6±33.4	25569.1±429.4	3724.7±520.2	39785.4±744	5211.2±255.7	8369.5±1290.4	5227.6±3124.1	4063.5±1938.1
creatine	246.4±111.8	455.1±128.9	778.8±170.2	1024.9±231.9	832.4±85.6	1322.5±80.4	689.4±335.7	867.6±203.9	267.8±76.7	405.6±56.8	838±8.2	1582.5±7.9	319±99.8	422.6±85.1	374.3±54.9	954±46.7	2782.8±420.4	4264.6±420.2	70.5±99.4
creatinine+phosphocreatine	997±80.5	1813.1±53.5	1831±153.4	2233±276.6	2852.5±67.4	2225.7±193.8	2468.3±363.5	1840.3±238.8	1014.3±69.3	1871.1±98.1	2011.7±18.6	1474.7±16.5	993.5±97.1	1253.3±145.5	1215.1±77.8	1681.6±99.9	2752.3±561.8	3227.2±779.4	1475.3±94.7
dimethylamine	60.6±11.5	111.9±9.6	97±4.9	115.8±13.3	286.8±55.8	221.8±3.1	88.8±22.4	91±13.8	53.3±4.8	102.8±8.8	155.9±9.4	140.4±7	218.8±36.2	152.3±44.7	88.3±12.2	105.9±2.4	274.9±50	427.8±365.2	141±91.3
methylamine	13.1±9.2	14.5±4.5	10.9±11.4	17.9±4.9	33.2±3.2	27.5±2.8	8.5±8.6	15.4±8.3	15.7±6.3	9.2±4.7	22.6±2.2	16.6±7.2	5.2±3.2	4.2±4.9	9.4±4.8	7.3±3	50.9±11.8	63±14.3	9.1±7
trimethylamine	14.1±7.3	52.7±15.7	32.3±10	46.6±5.1	155±49.8	70.9±20.7	19.4±17.3	52.2±9.6	8.5±7.7	63.1±14.8	61.5±5.4	14.9±8.4	26.5±6.4	40.4±9	25.2±7	44.3±1.8	31.4±12.2	32.7±31.7	3.9±7.1
**Vitamins**																			
ascorbate	1987.9±85.5	3569.6±474.9	2035.4±410.4	2549.8±305.4	1351.7±221	2401.2±124.7	2732±567.2	2443.3±180.7	1995.1±76.4	3446.5±191.5	2220±124.7	2664.7±48.3	2360.6±282.1	2251.7±188.3	2173.2±369.8	2610.6±44.6	721±354.4	600.6±44.4	2006.6±369.4
niacinamide	59.2±7.1	30±19.4	107.2±13.1	87.3±15.3	56.4±11.8	68.9±9.1	93.9±16.9	94.4±70.4	68.1±19.4	32.7±14.5	78.3±10.5	133.9±8.8	76.3±6.3	78.2±7.7	82.8±5	8.8±7.6	15.8±11.6	18.1±8.8	8.3±6.6
**Carbohydrates and sugars**																			
2'-fucosyllactose	0±0	0±0	0±0	0±0	0±0	0±0	1329±1576	0±0	0±0	0±0	1797±552	306±58.6	2517±95.02	313±3.67	0±0	0±0	0±0	0±0	3923.3±3015.2
3-fucosyllactose	3721±237	5949±323	0±0	0±0	0±0	0±0	2443±729	0±0	3854±170	6578±702	0±0	3051±65	0±0	0±0	3803±142	0±0	0±0	0±0	1931.3±2216.1
arabinose	20.7±29.5	24.7±42	35.6±36.4	95.5±129.8	159.5±29.5	163.7±56.2	2.8±5.9	203.3±61.3	17.6±10.2	12.6±28.1	132.6±229.7	0±0	0±0	112.4±8.2	0±0	161.3±10.8	25.6±41.8	0±0	124.4±248.9
fucose	613.3±96.7	2995.6±162.7	909.6±91	1249.6±242.5	2700.2±83.8	2563.1±272.8	1154.1±451.6	892.6±472.1	581.7±131.6	2999.3±204.6	843.1±8.2	510.8±22.8	1410.5±107.4	2385.3±45	741.9±75.1	1621.9±327.9	272.5±227.1	858.6±714	32±40.4
Fucosyl-α-1,3-N-acetylglucosamine	295.3±13.3	450.1±21.8	506±28.9	499.5±100.8	273.9±38.6	269±40	335.3±19.8	404.1±53.1	298.1±19.8	417.4±33.1	344±58.9	389.6±11.9	247±46.5	317.3±27.1	332.2±43.9	419.4±1.4	185.8±63.8	144.2±12.9	2284.1±1488.8

Fucosyl-α-1,4-N-acetylglucosamine	225.4±7.4	385.1±86.7	303±43.7	386.5±161.2	132.1±89.6	106.7±79.3	235.8±41.7	312.1±113.2	228.3±13.4	362.9±93.4	237±105.6	192.6±24	145.2±49.7	192.2±19.2	233.3±109	239.8±17	85.5±94.3	169.1±53.3	4168.5±1523.7
galactose	2297.9±159.7	4053.7±88.1	4251±425.7	2801.3±914.7	1955.9±80.3	1735.6±111.2	4383.5±669.8	1648.9±165.4	2260.5±167.2	4193.5±162.5	4489±80.5	2895.9±41.8	2121.1±43.4	2353±72.8	2499.3±172.8	1308.8±14.8	46329.4±54160.5	700.7±119.5	1142.5±294.9
glucose	11355±1007.8	16645±1737.9	0±0	0±0	0±0	0±0	2624.2±2849	0±0	11317±361.8	16179.7±1254.3	0±0	15771.5±279.5	0±0	0±0	9318.8±324.7	0±0	74162±87349.8	0±0	0±0
lactose	289695.3±3864	335319.8±10438.1	208143.4±10272.5	252418.3±9312.1	331919.9±23037.9	333311.9±7140.1	303521.9±13587	246119±22915.2	291475.8±3928.4	332399±9041.9	291497.5±315.4	190988.9±165.1	285392.7±5354.5	326262.8±1496.1	177372.5±4839.6	237641.7±2203.6	112052.1±105111.7	169632.7±7726	300481.5±6423.9
lactulose	828.7±104.9	3654±138.8	718±123.2	1131±275.1	2782.5±77.8	2605.9±295.1	1232±550.5	903.2±480.7	823.9±149.5	3677.4±146.4	584.9±12.9	413.8±7.1	1392.1±131.6	2513.2±35.1	956.7±53	1600.8±380.3	138.9±166.9	1022.7±614.4	0±0
maltodextrin	1148±135.1	1203.5±184.1	134391.7±8443.6	68367.9±5771.5	0±0	1476.7±94.8	5989.3±6932.4	52376±7304.1	1124.5±130.5	1247.8±502.4	12700±32	83712.5±1408.5	2040.4±206.8	2644.3±98.8	89846.4±5302.2	113508.1±100.9	0±0	0±0	3109.6±2362.7
mannose	28.7±5.5	63.9±9.6	53±8.6	46.3±10.1	62.4±7.6	66.5±11.4	59±16.2	35.6±6.5	26.2±11	62.2±5.1	59.3±9.8	36.3±9	48±6.8	65±14.5	33.7±4.2	34.1±4	62.5±22.5	10.9±12.5	26.8±10.7
N-acetyl carbohydrates	10746.9±255.2	10775.8±627.8	16140.4±1025.5	17062.8±1408.5	14834.2±950.5	13769.9±474.6	18319.3±2815.1	10161.4±2718.1	10980.6±411.1	10269.1±710	24784.1±320.3	13139.6±421.6	17173.9±259.6	13397.2±248	11073.3±490.5	9506.2±51	17330.4±682.2	13523.7±3521.6	79389.8±38608.9
raffinose	3.8±5.9	33.2±30.7	4248.2±438.5	2267.6±246.4	0±0	20.7±10.5	202.5±232.6	2713±321.9	5.9±13.3	22.1±14.9	309±30.2	2454.4±36.2	12.1±17.2	24.6±21.4	2607.6±170.8	3338.2±97.2	0±0	0±0	4.2±8.5
sucrose	0±0	0±0	0±0	0±0	0±0	0±0	0±0	77643.3±1850	0±0	0±0	0±0	99624.5±410	0±0	0±0	0±0	0±0	0±0	0±0	0±0
UDP-galactose	3.7±8.3	10.1±20.5	0.8±1.8	1.7±3.8	0±0	0±0	0.1±0.2	1.9±3.8	3.3±7.3	0±0	0±0	0±0	3.1±6.1	2.8±4.8	0±0	0±0	2.8±6.8	415±132.2	0±0
UDP-glucose	1.8±4	0.7±1.7	5.3±5.3	2.1±4.7	4.9±6	13.2±14.1	10.5±11.3	7.7±8.9	5.4±5.4	4.4±7.6	9.3±5	3.4±5.8	6.6±11.9	5.9±5.4	3.8±7.3	0.9±1.1	10.2±14.7	80.4±43.1	7.5±3.4
**Energetic compounds**																			
acetyl-carnitine	4.2±3	7.4±8.2	21.6±4.3	28±5.2	8.4±5.2	11.7±3.5	20.3±6.1	13.3±4.9	4±2.3	2.5±3.1	16.3±5.3	20.3±9.5	7.8±3.8	7.8±2.4	12.8±3.2	20.7±2.7	20.3±5	9.7±7.5	18.9±13.6
carnitine	502.2±379.8	826.2±296.5	1612±153.6	1507.5±174.5	1294.3±73.2	964.4±112.9	1949±920.3	948.6±112.8	350.2±133.3	978.8±184.4	1069.9±33.3	817.5±10.9	556.2±99.7	625.1±48.9	338.9±3	797.2±60.8	2125.2±707.8	10172±846.7	12280±4176.3
glycerophosphocholine	2022.9±298.5	5422.4±437.7	11180.1±873.9	9517.7±858.7	7482.4±904.7	7542.1±738.2	12900.4±991.1	6281.1±842.7	2062.5±271.6	5386.5±342.4	14511.5±349.4	6898.2±20.2	5344.7±320.2	7383.2±48.6	3553.5±313.2	5243.7±120.8	11187.9±1875.7	3457.6±2396.4	6243.7±2131.3
**Short chain fatty acids (SCFA)**																			
acetate	337.8±35	493.5±26.3	711.9±115.1	820.2±	1017.7±32.4	793.7±	859.6±	410.5±	326.1±40	536.7±	869.4±	603±7.7	446.5±78	603.9±	552.5±	843.9±	659.3±317.1	534.1±	108±115.5

				216.9		66.4	216.1	142.5		20.6	12.6			137.3	66.7	56.3		111.3	
butyrate	2.4±5.3	2.9±6.5	11.7±16.7	10.8±15.5	59.7±48.1	51.9±20.4	12.6±19	27±33.3	2.9±6.5	2.7±3.8	23.2±28.3	43.1±20.1	29.4±23.7	43.2±13.3	10.5±10.2	61.6±4.3	105.1±14.3	0±0	127±170.4
**Nucleotides and derivatives**																			
inosine	32.2±6.8	6.9±2.6	9.4±8.8	8.4±7.3	5.9±5	9.9±7.1	35.8±6.6	7.6±6.2	34.7±7.1	8.2±9	23.9±4.6	2.1±2.1	9.2±6.1	8.6±3.5	15.5±7.6	1.3±1.5	8.5±6.7	73±21	2.9±3.4
uridine	27.8±6.5	8.2±4.8	9.6±7.6	20±9	17.8±13.2	14.2±9.7	35.3±10.3	17±11.1	29.7±10.6	15.2±12.7	46.5±23	9.3±7.1	28.1±5.2	19.6±7.3	17.9±5.4	7.7±2.2	18.1±11.6	234.7±21.2	16±26.5
orotate	217.3±20.9	428.2±65	211.3±16.2	284.1±5.6	476.3±43.1	693.6±35.9	233.2±25.3	419.3±36.3	216.4±56.4	413.5±53.6	321.5±19.8	563.8±15	307.2±61.6	296.3±25.3	247.3±21.3	436.8±31.3	824.4±54.9	142.3±46.1	22.7±5.3
**Other compounds**																			
acetone	137.6±12.9	219.7±32.7	120.8±14.1	117.4±16.2	295.5±13.9	195±29.3	204.9±76.6	160.6±122.3	136.7±17	235.9±45.6	289.6±37.7	239.1±2.3	273.5±33.6	274.5±6.6	294±34.4	261.3±2.4	148.5±99	73.1±26.3	48.9±6.2
dimethyl sulfone	91.5±17.6	138.8±25.5	175.8±9.5	256±35.8	399.5±33.6	437.7±33.5	216.4±22.3	162.4±70.6	82.9±34.7	120.5±31.9	299.3±2.8	209.6±4.7	418.8±106.1	319±145.8	100.1±12.9	128.1±35	607.4±127	634.1±93.4	163.1±13.1
ethanol	0±0	0±0	0±0	0±0	0±0	0±0	0±0	0±0	0±0	0±0	0±0	0±0	0±0	0±0	0±0	0±0	47.6±52.4	1.4±2.9	0±0

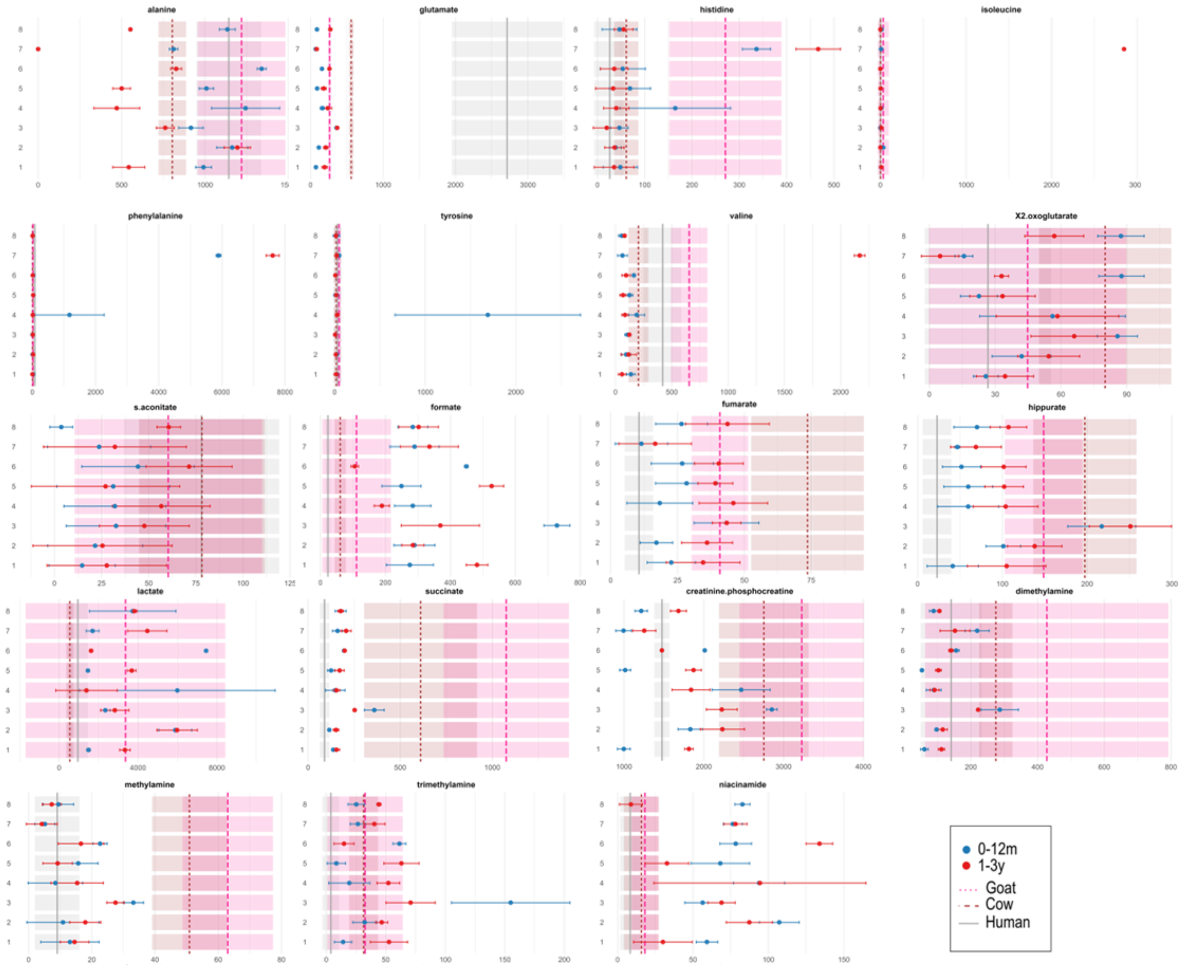

Figure S10. Dumbbell plot of the amino acids, organic acids, amine and derivatives and vitamins. The plots were constructed using the means and standard deviations calculated for each milk type group. On the y-axis, we have the 8 brands of artificial formulas. The blue dots represent the 0-12m type for each FM (formula milk), while the red dots represent the 1-3y type. On the x-axis, we find the calculated peak areas for each signal assigned to a specific metabolite, i.e., the relative intensities of each metabolite. The vertical lines on the plot indicate the group means for goat milk (dashed pink line), cow milk (both lactose-free and conventional, dashed brown line), and human milk (solid gray line). Standard deviations (SD) were also calculated for these and are represented by shaded bands in the same color as the corresponding line. This was done to better describe the trends of the different groups.

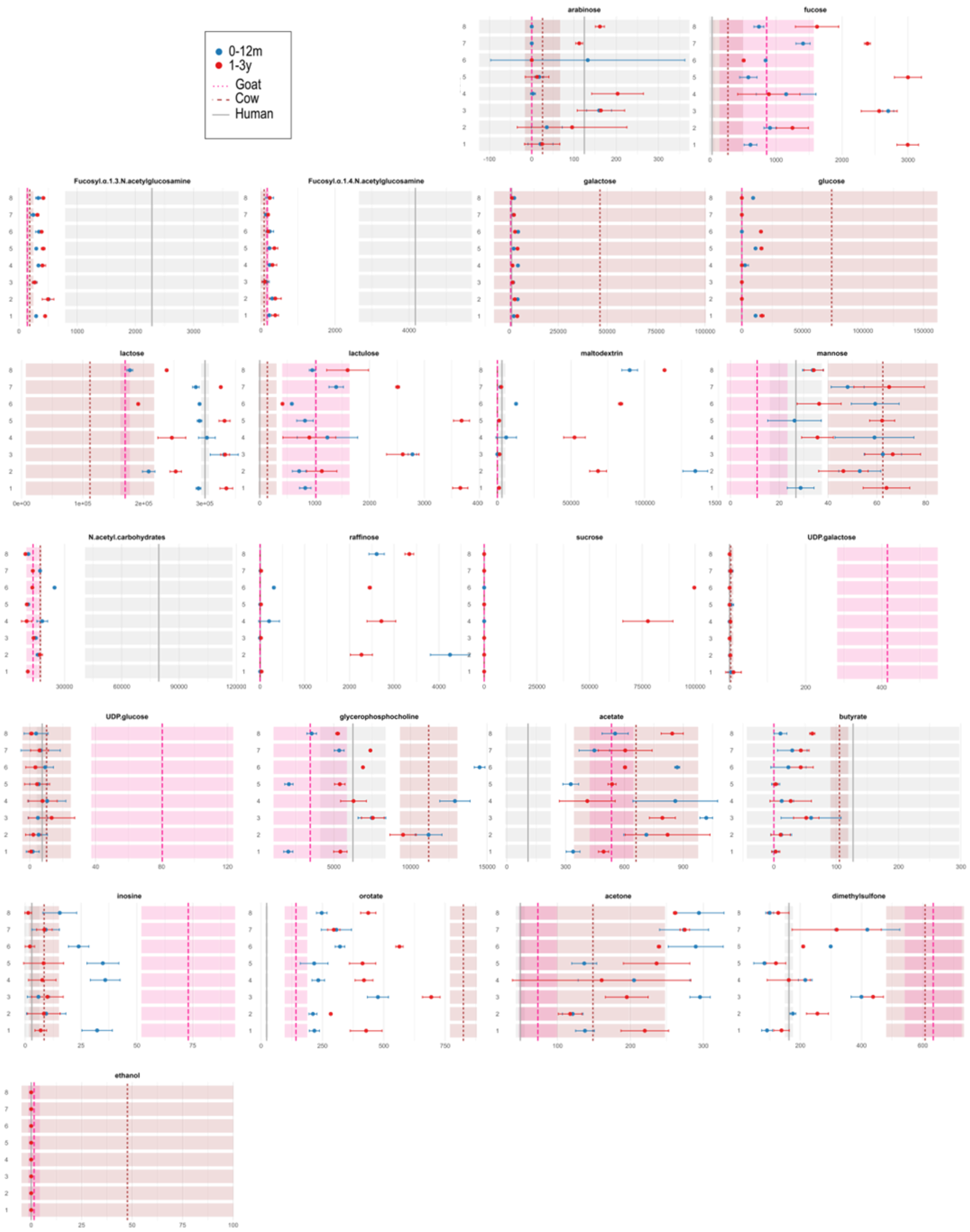

Figure 11. Dumbbell plot of the carbohydrates and sugars, energetic compounds, short chain fatty acids, nucleotides and derivatives and other compounds. The plots were constructed using the means and standard deviations calculated for each milk type group. On the y-axis, we have the 8 brands of artificial formulas. The blue dots represent the 0-12m type for each FM (formula milk), while the red dots represent the 1-3y type. On the x-axis, we find the calculated peak areas for each signal assigned to a specific metabolite, i.e., the relative intensities of each metabolite. The vertical lines on the plot indicate the group means for goat milk (dashed pink line), cow milk (both lactose-free and conventional, dashed brown line), and human milk (solid gray line). Standard deviations (SD) were also calculated for these and are represented by shaded bands in the same color as the corresponding line. This was done to better describe the trends of the different groups.

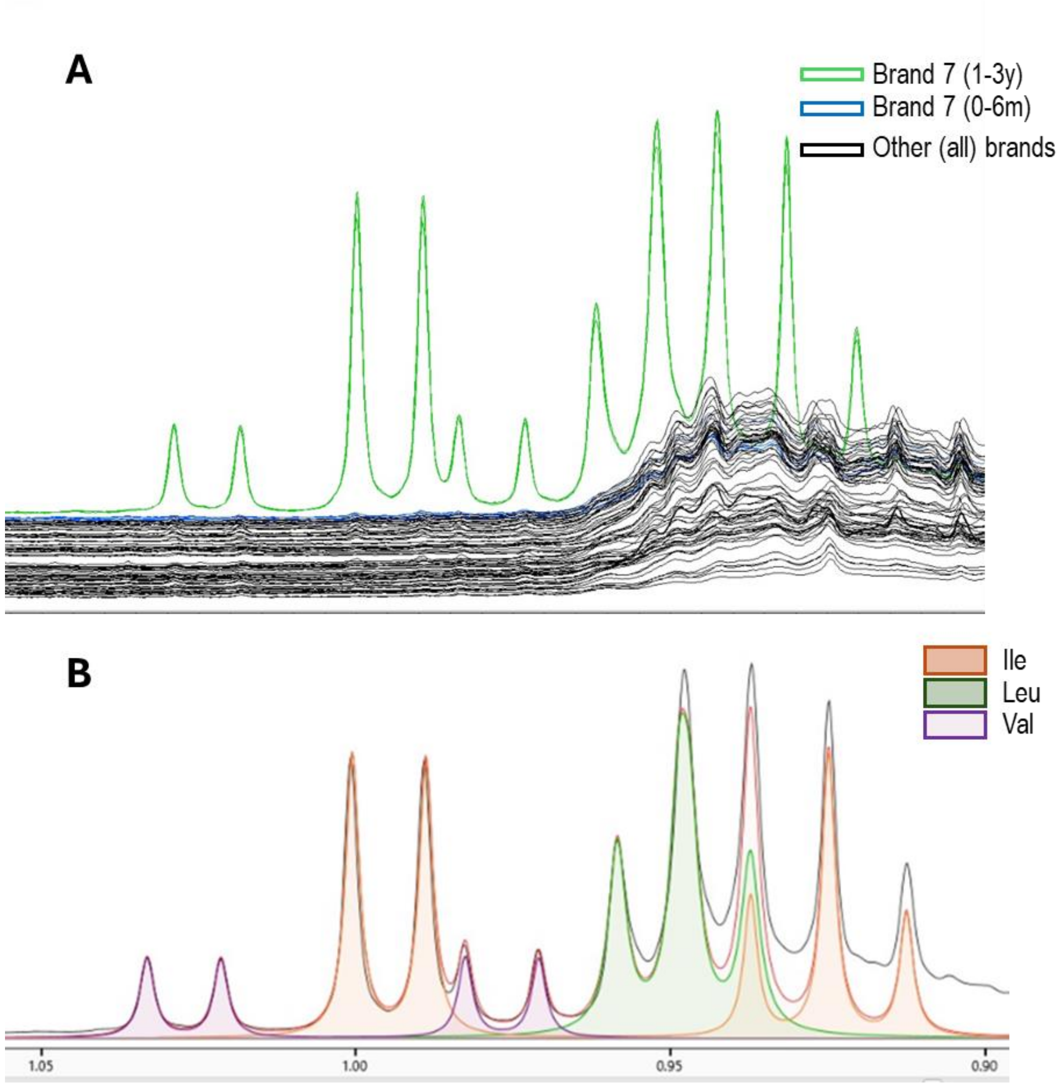

Figure S12. The figure displays the signals of isoleucine, leucine, and valine for Brand 7, formulation 1-3y. Panel A shows the spectra in green (Brand 7, 1-3y formulation), blue (Brand 7, 0-12m formulation), and black (all spectra from other brands). Note that no spectra from cow, goat, or human milk are included in this comparison. This panel highlights the significantly higher signals of these amino acids in Brand 7, 1-3y formulation. Panel B presents the peak assignments for isoleucine (orange), leucine (green), and valine (purple), performed using Chenomx software. The x-axis displays chemical shifts in ppm.

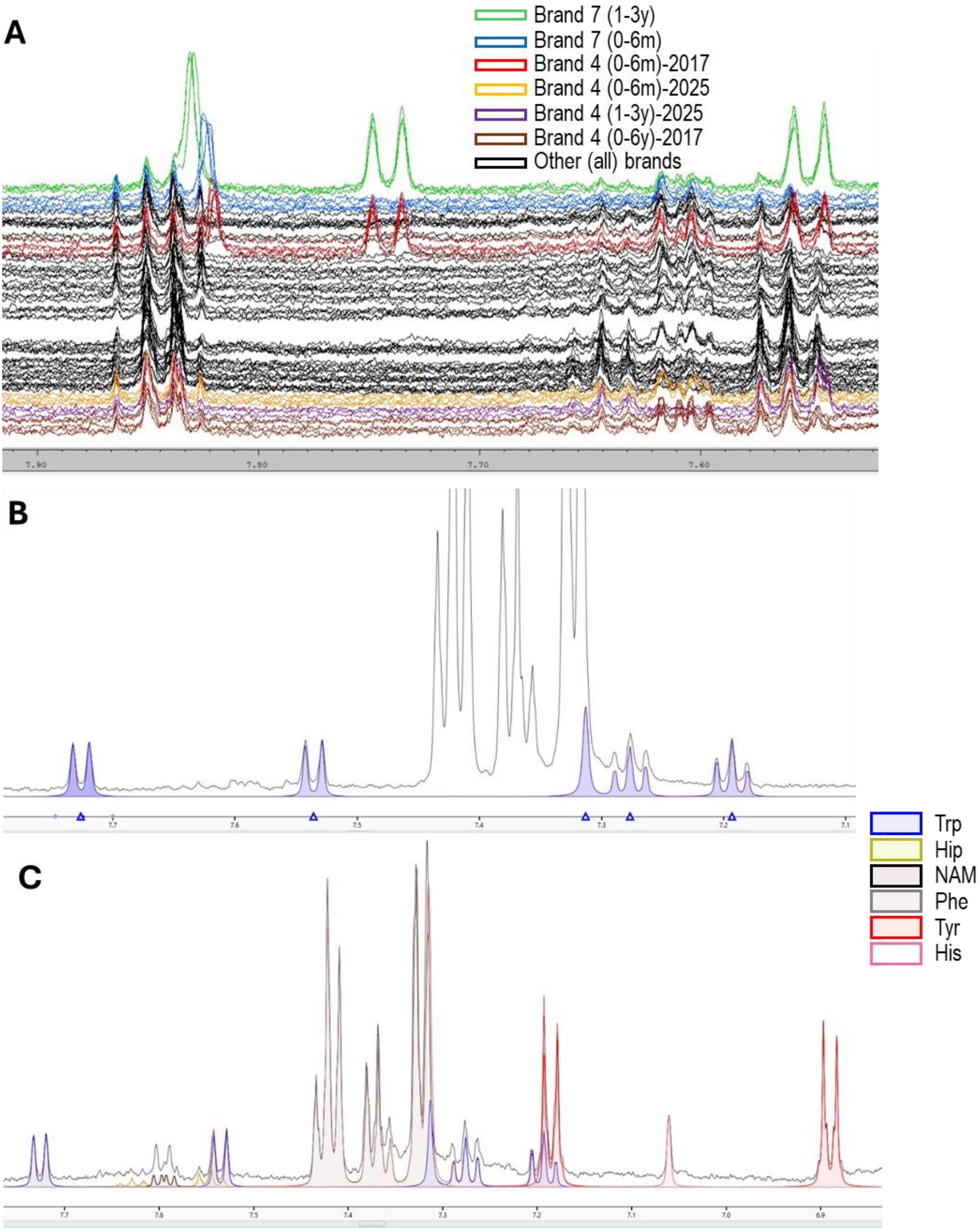

Figure S13. The figure displays the characteristic tryptophan (Trp) signals for two specific formulations: Brand 7 (1-3y) and brand 4 (0-12m) from the previous sample collection. Panel A (^{1}H NMR spectra) shows the distinct Trp signal at 7.75 ppm (d, -CH), which is unique to these two brands and formulations. Panel B presents the Trp peak assignment for Brand 7 (1-3y). Panel C shows the assignments for tryptophan (Trp), hippurate (Hip), niacinamide (NAM), phenylalanine (Phe), tyrosine (Tyr), and histidine (His) in Brand 4 (0-12m), sampled and acquired in 2017.
